# Computational Characterization of Zr-Oxide MOFs for Adsorption Applications

**DOI:** 10.1021/acsami.2c13391

**Published:** 2022-12-14

**Authors:** Rama Oktavian, Raymond Schireman, Lawson T. Glasby, Guanming Huang, Federica Zanca, David Fairen-Jimenez, Michael T. Ruggiero, Peyman Z. Moghadam

**Affiliations:** † Department of Chemical Engineering, University College London, London WC1E 7JE, U.K.; ‡ Department of Chemical and Biological Engineering, The University of Sheffield, Sheffield S1 3JD, U.K.; § Department of Chemistry, University of Vermont, Burlington, Vermont 05405, United States; ∥ Department of Chemical Engineering & Biotechnology, University of Cambridge, Philippa Fawcett Drive, Cambridge CB3 0AS, U.K.

**Keywords:** Zr-oxide MOFs, structure−property relationships, CO_2_ adsorption, carbon capture, DFT calculations, augmented reality

## Abstract

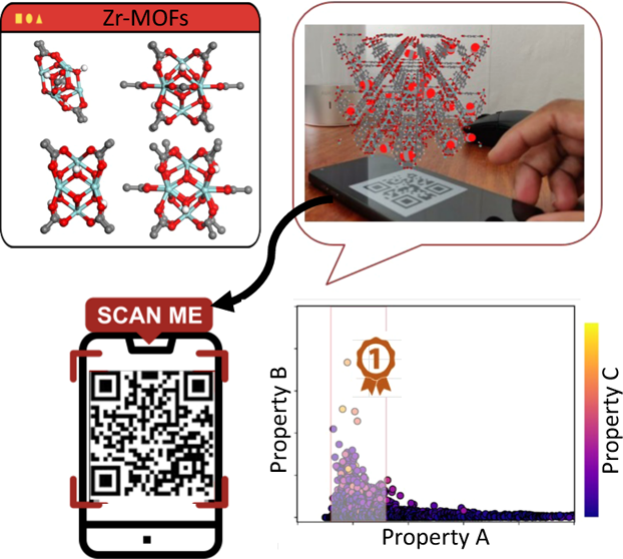

Zr-oxide secondary building units construct metal–organic framework (MOF) materials with excellent gas adsorption properties and high mechanical, thermal, and chemical stability. These attributes have led Zr-oxide MOFs to be well-recognized for a wide range of applications, including gas storage and separation, catalysis, as well as healthcare domain. Here, we report structure search methods within the Cambridge Structural Database (CSD) to create a curated subset of 102 Zr-oxide MOFs synthesized to date, bringing a unique record for all researchers working in this area. For the identified structures, we manually corrected the proton topology of hydroxyl and water molecules on the Zr-oxide nodes and characterized their textural properties, Brunauer–Emmett–Teller (BET) area, and topology. Importantly, we performed systematic periodic density functional theory (DFT) calculations comparing 25 different combinations of basis sets and functionals to calculate framework partial atomic charges for use in gas adsorption simulations. Through experimental verification of CO_2_ adsorption in selected Zr-oxide MOFs, we demonstrate the sensitivity of CO_2_ adsorption predictions at the Henry’s regime to the choice of the DFT method for partial charge calculations. We characterized Zr-MOFs for their CO_2_ adsorption performance via high-throughput grand canonical Monte Carlo (GCMC) simulations and revealed how the chemistry of the Zr-oxide node could have a significant impact on CO_2_ uptake predictions. We found that the maximum CO_2_ uptake is obtained for structures with the heat of adsorption values >25 kJ/mol and the largest cavity diameters of ca. 6–7 Å. Finally, we introduced augmented reality (AR) visualizations as a means to bring adsorption phenomena alive in porous adsorbents and to dynamically explore gas adsorption sites in MOFs.

## 1 Introduction

Metal–organic frameworks (MOFs) are now a well-established generation of porous materials, which are formed by extended coordination networks of metal clusters and organic building blocks. MOFs can be readily tailored to produce thousands of materials with a vast range of pore sizes, shapes, and chemistries with promise for numerous applications in specific gas storage and separation.^1−7^ Indeed, we have already identified a staggering ca. 100,000 already-synthesized MOFs in the Cambridge Structural Database (CSD) as of January 2020 (2020.0 CSD release).^8−10^ Of all the identified MOF materials in the CSD, ca. 10,000 are porous (i.e., materials with pore-limiting diameter (PLD) > 3.7 Å), which can be explored for adsorption applications. In our recent work, we developed an array of algorithms to classify different families of porous MOFs based on some of their most well-known metal secondary building units (SBUs).^9^ Clearly, such databases and classifications create excellent opportunities for in silico screening practices for targeted exploration of MOFs for gas adsorption.^9^ However, in this context, a crucial and often overlooked aspect of MOFs is their ability to withstand exposure to industrial processes for gas sorption. With this idea in mind, here, we focus on the computational characterization of one of the undoubtedly key families of stable MOFs: structures containing Zr-oxide SBUs. We expect that this study will guide experimental and theoretical researchers to carry out transformative research on this promising class of MOFs for novel adsorption technologies.

Zr-oxide MOFs have great potential for gas adsorption and separation applications owing to their porosity, regenerability, and good chemical and physical stability properties.^11−13^ The high oxidation state of Zr generates strong Zr–O SBUs, and the high connectivity with the organic ligands boosts their mechanical stability.^14,15^ Furthermore, most structures maintain their structural integrity at temperatures up to 450 °C and in aqueous or acidic conditions.^12,13,16−18^

Given the large number of existing MOFs, including those containing Zr-oxide nodes, quite a few high-throughput computational protocols have been developed to screen and identify top-performing materials before synthesis and experimental testing in the laboratory.^19−23^ Predominantly, theoretical approaches combining density functional theory (DFT) and grand canonical Monte Carlo (GCMC) calculations have successfully predicted the gas adsorption properties of MOFs. The accuracy of such simulations relies on the force field parameters that describe adsorbate–MOF and adsorbate–adsorbate interactions. In addition to van der Waals interactions, for polar and quadrupolar adsorbates, it is essential to take into account the electrostatic interactions between the adsorbate and the MOF atoms. Such interactions are normally described by assigning partial charges to framework atoms. Different methods have been developed to calculate MOF’s atomic charges, for which significant variations in adsorption predictions could arise.^24−29^ Popular methods include semi-empirical approaches, such as charge equilibration methods^30^ and those based on bond connectivity^31^ that require no electronic structure calculation, or charge assignment approaches based on quantum mechanical calculations, such as CHarges from ELectrostatic Potentials using a Grid-based method (ChelpG),^32^ density derived electrostatic and chemical (DDEC),^33^ and repeating electrostatic potential extracted atomic (REPEAT),^34^ to generate electrostatic-potential-derived atomic charges. A number of studies have compared the sensitivity of gas adsorption predictions with respect to the method used to assign partial atomic charges. In an outstanding contribution, Nazarian et al.^35^ compared atomic point charges derived from EQeq and the DDEC approaches for the adsorptive removal of *tert*-butyl mercaptan from natural gas and found a significant difference in predicted adsorption selectivity depending on the charge assignment method. In general, the DDEC approach reproduces the electrostatic interactions outside the van der Waals radius of atoms, and this is especially important for gas adsorption simulations. Similar to the conclusions of this work and others,^24^ the general consensus is that DFT methods provide more accurate partial charge predictions and therefore are more reliable for gas adsorption simulations especially in the low-pressure regime-Henry’s region in the isotherm, where adsorbate–MOF interactions dominantly determine the amount of the gas adsorbed and the shape of the isotherm.

Importantly, we note that none of the 2932 experimentally synthesized MOF structures in Nazarian et al.’s work are Zr-oxide MOFs, and given the importance of this class of materials, in the present work, we performed a systematic study to identify, characterize, and calculate the partial atomic charges for Zr-oxide MOFs present in the CSD MOF subset. Finally, we use this subset of structures in a high-throughput screening study to explore their capabilities for CO_2_ capture in flue gas conditions.

## 2 Results and Discussion

### 2.1 Zr-Oxide MOFs: Identification and Geometric Characterization

**Figure 1:**
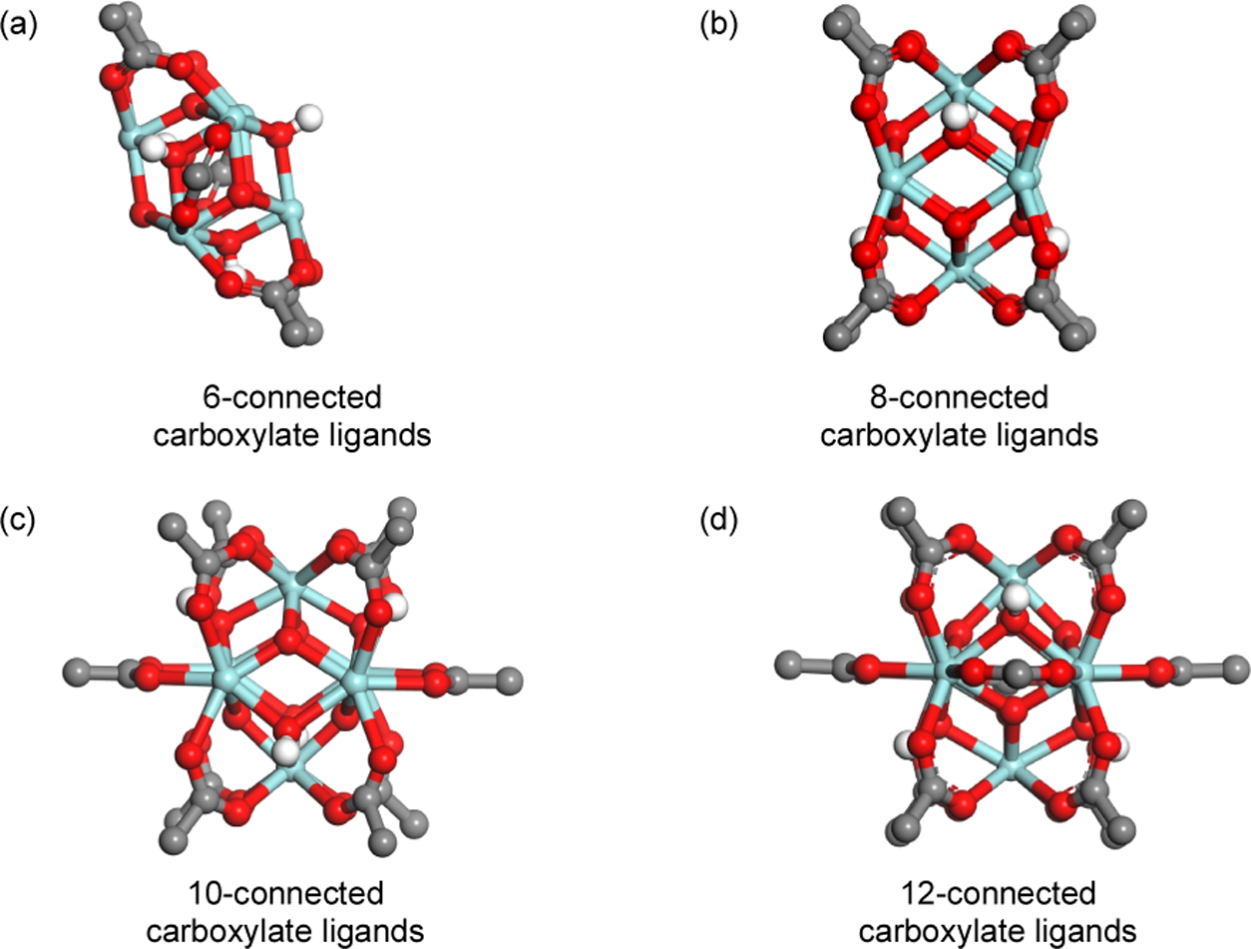
Three-dimensional (3D) representation of Zr-oxide secondary building units (SBUs) based on Zr_6_ clusters: (a) 6-connected carboxylates in PCN-224; (b) 8-connected carboxylates in PCN-222; (c) 10-connected carboxylates in DUT-67; (d) 12-connected carboxylates in UiO-66.

We used ConQuest, the CCDC’s primary software for searching structures, to develop seven search queries (Figure S1) to identify all MOF materials containing the Zr-oxide cluster from the CSD MOF subset. We successfully hit 102 structures among the existing ca. 100,000 MOFs (Figure 1).

**Figure 2:**
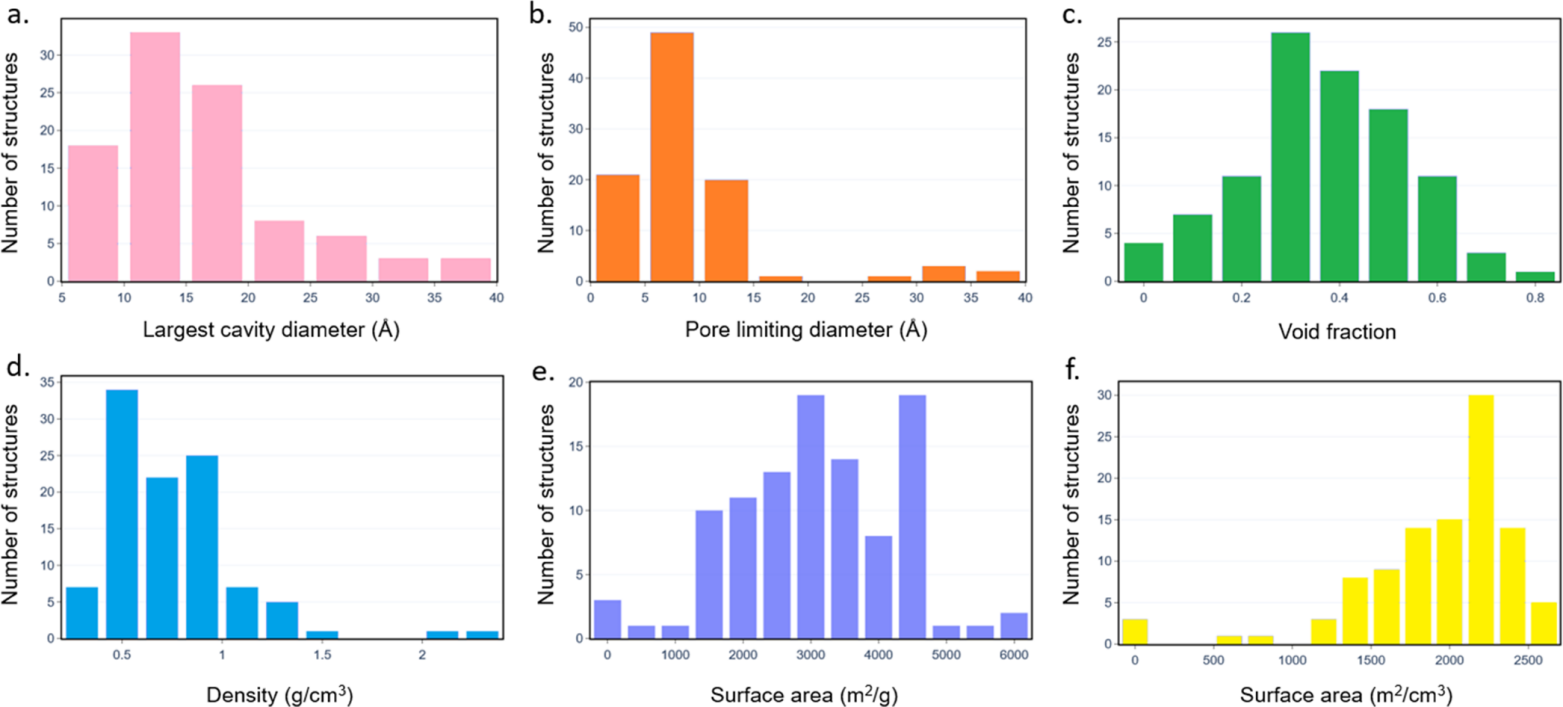
Histograms showing the calculated geometric properties of 102 Zr-oxide MOFs in the CSD MOF subset. (a) Largest cavity diameter, (b) pore-limiting diameter, (c) void fraction, (d) density, (e) gravimetric surface area, and (f) volumetric surface area.

After generating the subset of Zr-MOFs and fixing the position of OH groups for each structure,^36−39^ we calculated different geometric descriptors such as accessible surface area (ASA), largest cavity diameter (LCD), pore-limiting diameter (PLD), void fraction, and density using the Zeo++ software package.^40^Figure 2 shows the distribution of the geometric properties. While a wide range of physical properties is achievable in Zr-oxide MOFs, most structures are concentrated in regions with micropores with LCDs < 20 Å and void fractions of 0.3–0.5, possibly due to the commercial availability of shorter linkers such as benzene–dicarboxylic acid and the popularity of this range of pore size for gas storage and separation applications. Of all, 85% of structures have gravimetric surface areas between 1500 and 4500 m^2^/g, volumetric surface areas of 1500 and 2500 m^2^/cm^3^, and densities <1.5 g/cm^3^. The geometric and physical properties of all the Zr-oxide MOFs are tabulated in the Excel file in the Supporting Information.

**Figure 3:**
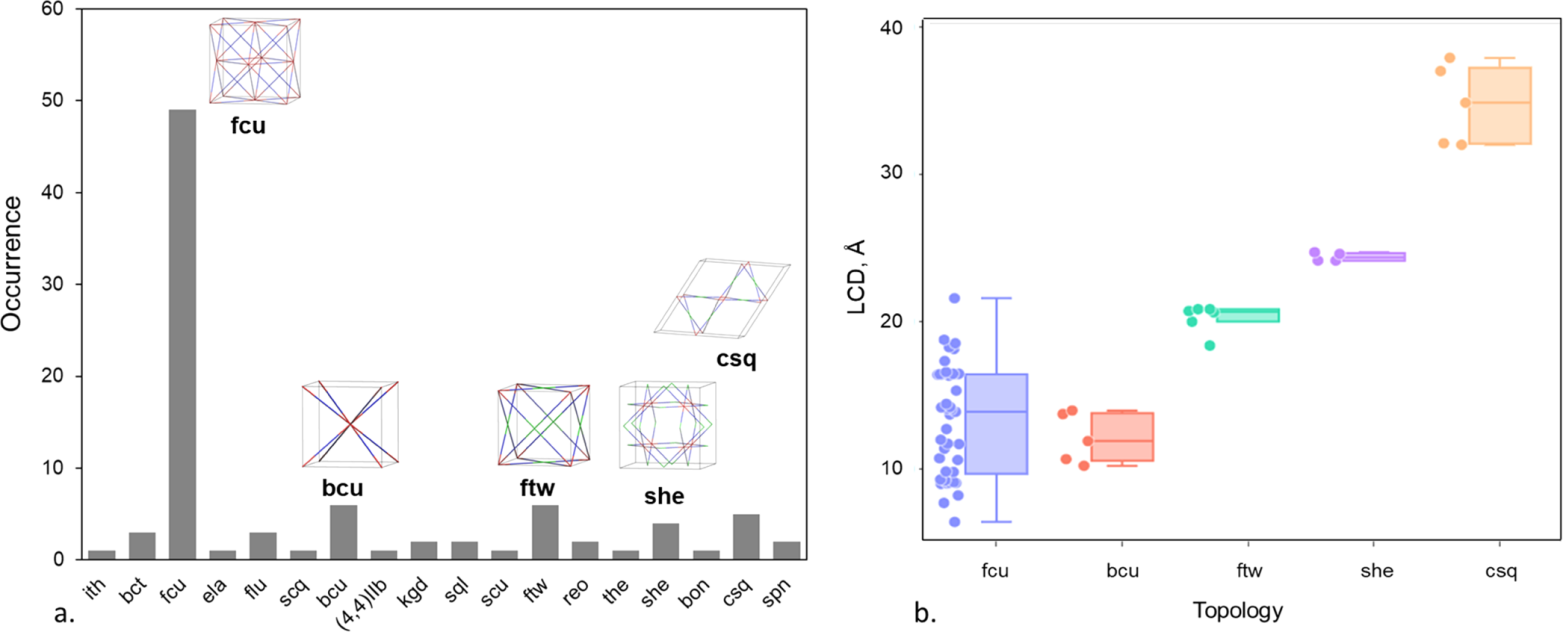
(a) Histogram for the distribution of topologies for Zr-oxide MOFs; top-five topologies are shown in the inset: Zr-oxide node is shown in red, linkers in blue, and the green represents organic nodes. (b) Distribution of the largest cavity diameter (LCD) in selected topologies.

The description of MOF topology is important as it provides the underlying connectivity of their building units.^41^ Different topologies can result in variations in the pore size and shape, as well as mechanical stability.^15,42^ We used CrystalNets^43^ to assign topologies to each structure using a single-node simplification approach. Of the total 102 materials, we assigned Reticular Chemistry Structural Resource (RCSR)^41^ topology identifiers to 100 structures, with one unknown topology and one topological type database (TTD) topology, which was obtained using ToposPro^44^ from the Samara Topological Data Center. Figure 3a shows the frequency of topologies for the structures in the subset with 17 unique three-character RCSR identifiers and one unique TTD identifier. A number of materials were assigned with two topologies; in cases where metal nodes are linked by porphyrins or derivatives thereof, the consequence of changing the ring size parameter in Topos’ cluster simplification method results in an alternative allocation of the connectivity of nonmetal nodes. The central porphyrin ring can be simplified into either four 3-connected nodes about the edges of the porphyrin structure or a single 4-connected node, resulting in the possible allocation of both **xxw** and **ftw** topologies, respectively. The result is subjective, but these structure types have been previously reported as **ftw** maintaining a single node at the center of the porphyrin linker. Additionally, the set contains 10 structures that consist of layers of interpenetrating material represented by two-dimensional (2D) **kgd** and 3D **fcu** nets. The five most frequently reported 3D periodic nets in this set are **fcu** with 49 structures followed by five structures for each **bcu**, **csq**, **she**, and **ftw** topology. Finally, a single 2D **sql** net was reported for the GOXZAW structure, which consists of layers of nonconnected 2D metal–organic sheets. Figure 3b shows the range of LCD values for the five most common topologies and shows that certain topologies may impose limits for structural descriptors including the pore size. LCDs range from 6.4 Å (**fcu**) to 37.9 Å (**csq**), varying dramatically from micropores to mesopores. Zr-oxide MOFs with **fcu** and **bcu** topologies exhibit micropores with LCDs < 20 Å. The structures with **ftw**, **she**, and **csq** topologies lie within the mesoporous region with LCD > 20 Å, where materials with **csq** topology contain the largest cavity diameter among all Zr-oxide MOFs.

### 2.2 N_2_ Adsorption; Brunauer–Emmett–Teller (BET) Area Characterization

The surface area of MOFs is arguably their most attractive feature and is typically measured or calculated as part of MOF porosity characterization via N_2_ adsorption. Here, we calculated N_2_ adsorption isotherms at 77 K for all 102 Zr-oxide structures via GCMC simulations and estimated the BET area following the consistency criteria suggested by Rouquerol et al.^45^ All N_2_ adsorption isotherms and details of the BET area calculations are reported in the Supporting Information (Section S3).

**Figure 4:**
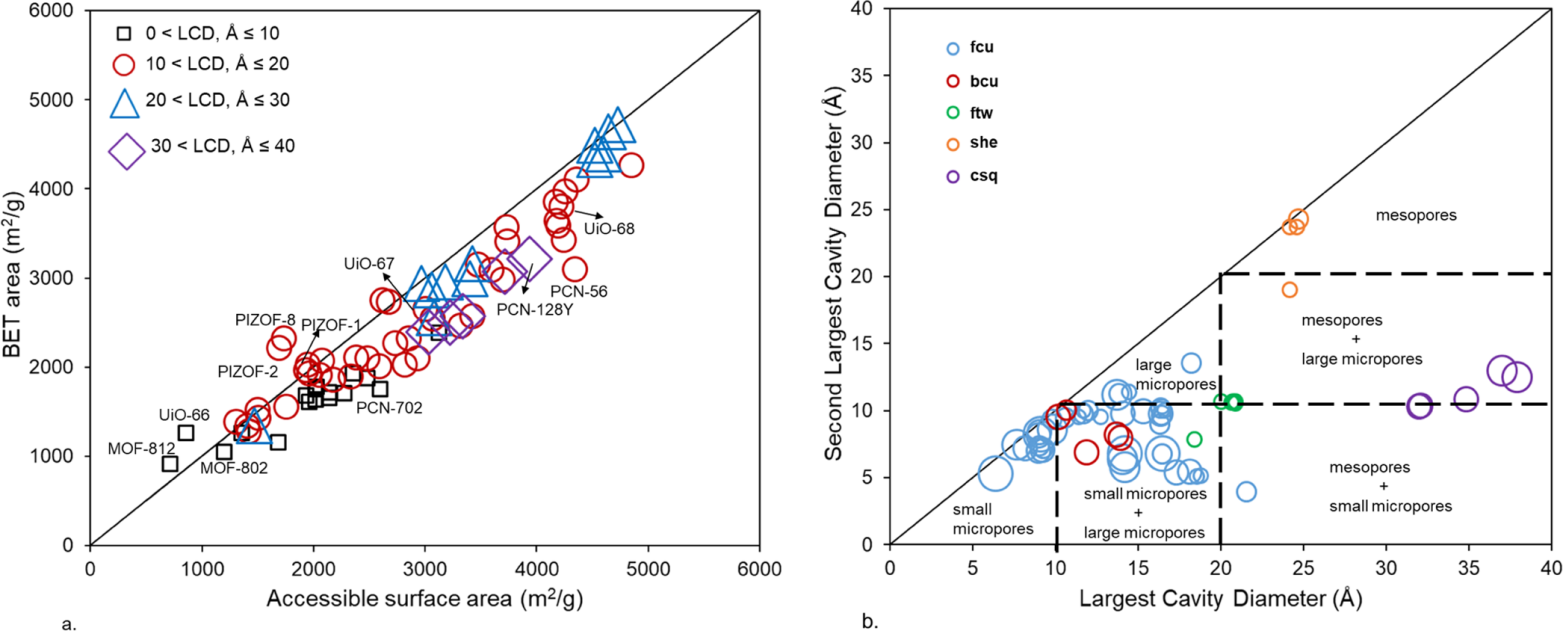
(a) BET area vs accessible surface area calculated for 102 Zr-oxide MOFs with various ranges for the largest cavity diameter (LCD); (b) relationship between the largest cavity diameter and the second-largest cavity diameter for the top-five topologies in Zr-oxide MOFs. The color represents the topology; the size of the data points corresponds to the proportional absolute deviation between the BET area and ASA.

Among all structures, 85 MOFs complied with the four so-called “BET consistency criteria”, while we had trouble finding the pressure range that would satisfy all four criteria for the remaining 17 structures. For these structures, after applying the first criterion, only one possible pressure range for BET calculations is eligible, meaning that the surface area calculated is insensitive to the remaining BET area criteria. Figure 4a shows the parity plot comparing BET area values with the accessible surface area (ASA) for structures with different LCDs. The results show that, for the majority of structures, irrespective of their LCD range, ASA calculations report higher values compared to the BET area. We note that ASA is calculated using the Zeo++ software, where a spherical probe with a radius of 1.86 Å (the kinetic radius of N_2_) was used. We also note that almost all Zr-oxide MOFs have multiple pores in their structures with some as small as ca. 3.5–5 Å, i.e., their pore size is similar to the kinetic diameter of N_2_ [see e.g., UiO-66, MOF-801, MOF-802, MOF-812, and PCN-702 pore size distribution (PSD) in the Supporting Information]. The presence of these small pores creates regions in the structure that may not be easily accessible to N_2_ molecules in GCMC simulations and therefore underestimating the monolayer loading.

According to the previous study performed by Gómez-Gualdrón et al.,^46^ BET calculations for MOFs that fulfill all four consistency criteria can still inaccurately characterize the true monolayer loading, specially for structures combining mesopores (*d* ≥ 20 Å) and large micropores (*d* = 10–20 Å). Figure 4b presents the distribution of LCD and the second-largest cavity diameter for the top-five topologies and highlights the deviation between the BET area and ASA, represented proportionally by the size of the data points. For the **fcu** topology, large deviations were seen for structures with small micropores (pore diameter <10 Å) and those combining large micropores and small micropores. This was also observed for MOFs with the **bcu** topology (20% deviation) where there are no structures with LCD larger than 20 Å and those structures with small micropores as their LCD. Zr-oxide MOFs with **ftw** and **she** topologies show low deviation (<10%) since they mostly contain LCD > 20 Å and no micropores as their second LCD. A significant deviation (20–40%) was observed for structures with the **csq** topology. These structures combine mesopores (*d* ≥ 20 Å) with a higher proportion compared to the large micropores (*d* = 10–20 Å). Such deviations could arise from how the Voronoi nodes are sampled in Zeo++ and the accessibility of tight regions of the pore to the nitrogen’s geometric probe model. We hope this analysis provides a benchmark for assessing the quality of Zr-oxide MOFs, for example, what is the upper limit of the surface area that can be achieved if the structural integrity is maintained or if the MOF is completely activated in the solvent removal process? This will, in turn, give information about the expected experimental adsorption performance of a MOF.

### 2.3 Atomic Charge Assignment and CO_2_ Adsorption Simulations

**Figure 5:**
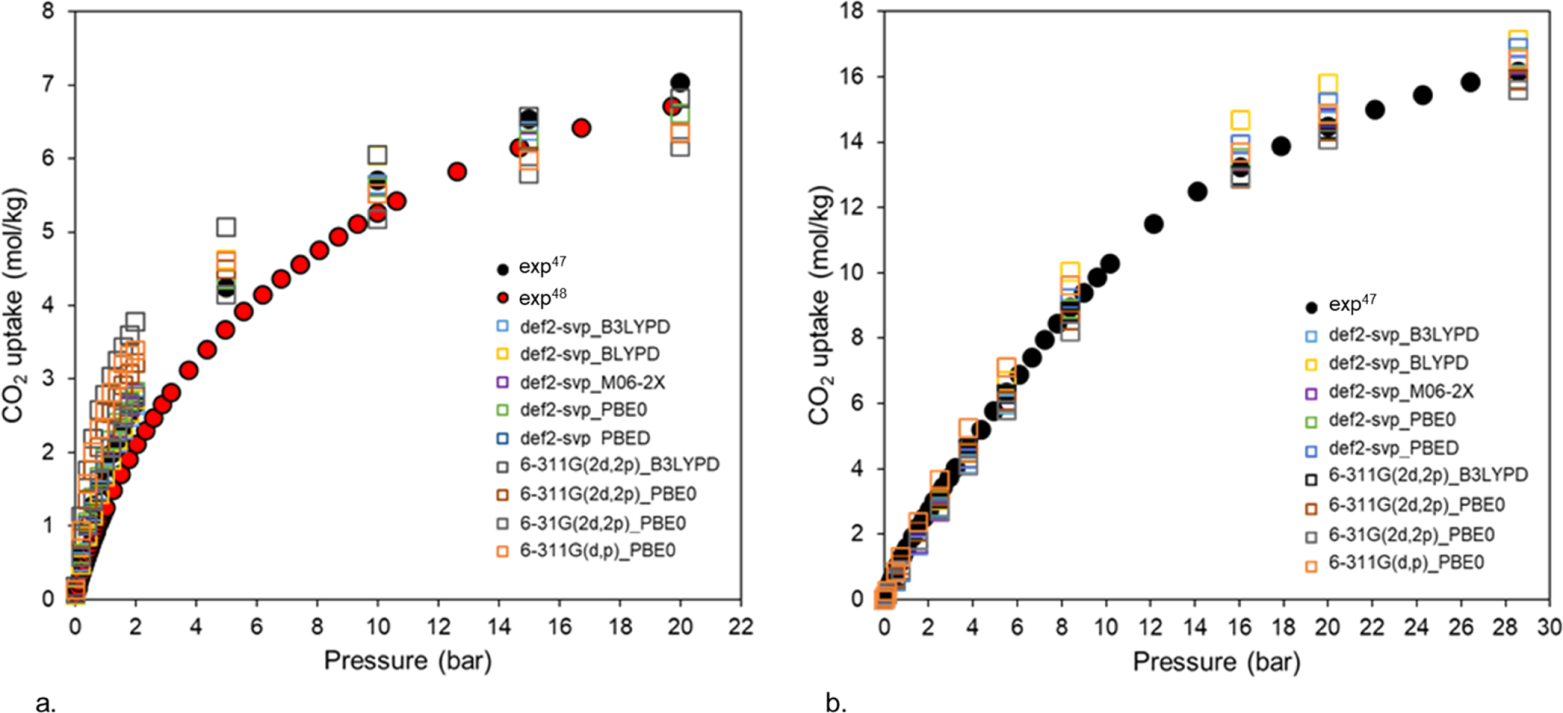
Simulated and experimental CO_2_ adsorption isotherms for (a) UiO-66 and (b) UiO-67 at 298 K. The isotherms were calculated using different DFT calculations for partial atomic charge assignment.

We also investigated the performance of Zr-oxide MOFs for CO_2_ capture. Before running the calculations in a high-throughput manner, we first compared the calculated adsorption isotherms with experimental data available in the literature. Given that CO_2_ has a high quadruple moment, its adsorption prediction highly relies on the electrostatic interactions with the framework. Therefore, it is important to take into account such interactions using electronic structure calculations such as density functional theory (DFT). DFT calculations can provide insight into the electrostatic potential energy surface, which can be tabulated as point charges on framework atoms. To study the performance of DFT calculations to predict partial atomic charges, we performed systematic periodic DFT calculations comparing 25 different combinations of basis sets and functionals. To do this, we first selected two prototypical Zr-oxide MOFs, namely, UiO-66 and UiO-67, where experimental CO_2_ adsorption isotherms were available. We then investigated the sensitivity of assigned point charges and the relevant CO_2_ adsorption isotherms for different DFT methods. Figure 5a,b shows the calculated CO_2_ adsorption isotherms obtained from various combinations of DFT functionals and basis sets and compares them with experiments for UiO-66 and UiO-67, respectively^47,48^ (Figures S4 and S5 present data for all combinations of DFT basis sets and functionals attempted). In general, the amount of CO_2_ adsorbed at high pressure and the shape of the isotherms are rather similar when electrostatic interactions are taken into account through different DFT calculations. For UiO-66, at low-pressure regime (ca. 1 bar), where the framework–CO_2_ interactions are dominant, the amount of predicted CO_2_ ranges between 1.7 and 3 mol/kg depending on the DFT method used. Such deviations are less prominent for UiO-67, a structure with larger pores, with CO_2_ uptake predictions ranging between 0.8 and 1.3 mol/kg at 1 bar. For both UiO-66 and UiO-67, the combination of PBE0 functional with the def2-svp basis set provides an excellent match with the experimental data, and therefore, we selected this DFT method to calculate partial atomic charges for the rest of the Zr-oxide MOFs (37 structures). The remaining calculations failed to converge to a suitable state or were too computationally expensive at this level of theory and thus were omitted.

**Figure 6:**
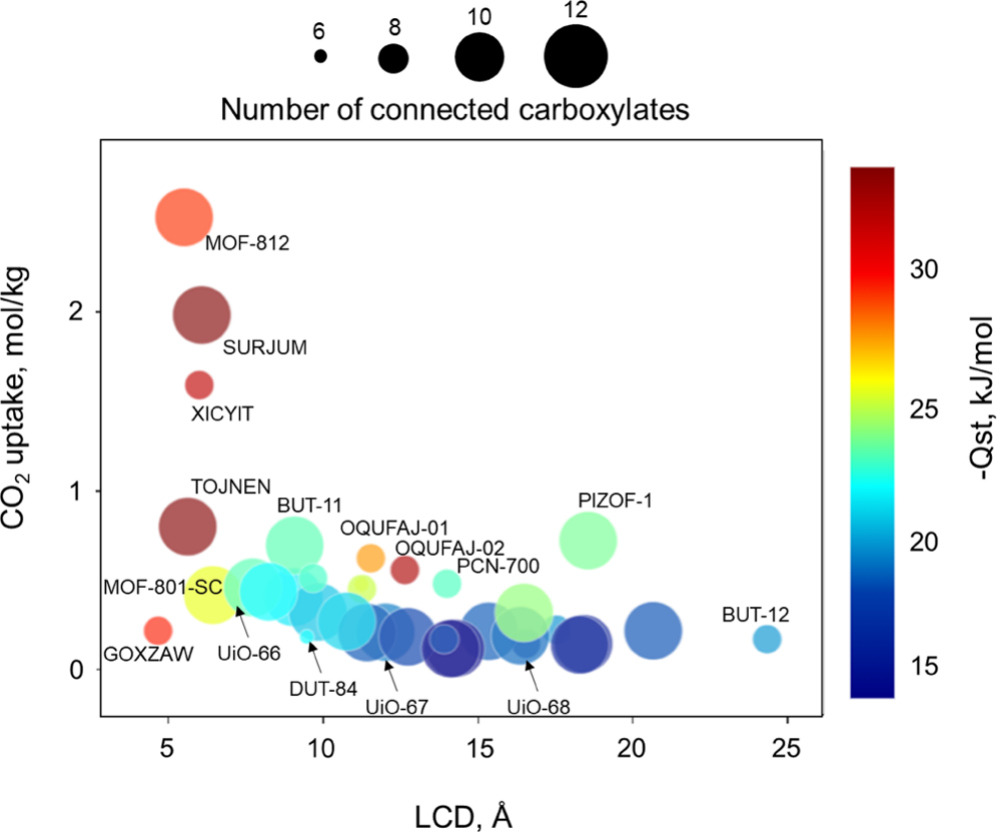
Structure–property relationships for CO_2_ capture in Zr-oxide MOFs at 0.15 bar and 298 K.

**Figure 7:**
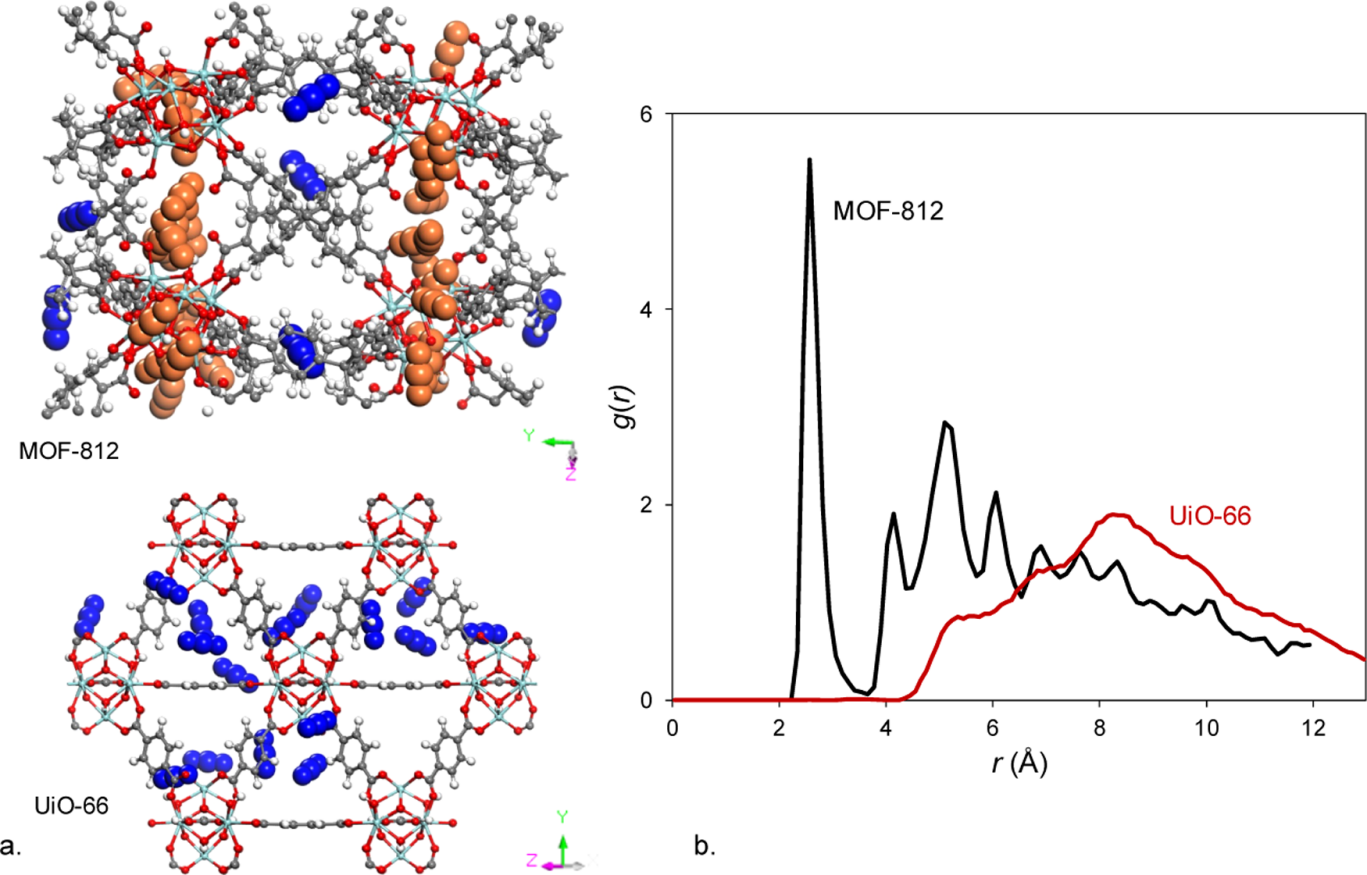
(a) CO_2_ adsorption snapshots in MOF-812 and UiO-66 simulated at 0.15 bar and 298 K. In UiO-66, CO_2_ molecules are adsorbed in small tetrahedral pores. In MOF-812, CO_2_ molecules sit preferentially in the space between the Zr-oxide nodes (orange vdW representation) in addition to the pockets created by the tetrahedral linkers (blue vdW representation). (b) Radial distribution functions between the Zr atom of the MOF and the O atom in CO_2_ molecules in MOF-812 and UiO-66.

After we calculated the partial atomic charges for the Zr-oxide MOFs, we performed high-throughput GCMC simulations to identify promising materials for CO_2_ adsorption. We targeted CO_2_ capture from flue gas in the post-combustion application at 298 K and 0.15 bar. Details of adsorption simulations are outlined in the Section 3. Similar to the adsorption of other gases, CO_2_ capture in Zr-oxide MOFs likely depends on different structural descriptors, including LCD, surface area, void fraction, structure density, as well as the heat of adsorption (*Q*_st_). To maximize CO_2_ capture performance, the combination of these descriptors must be optimized. Figure 6 shows the relationship between the amount of CO_2_ adsorbed at 0.15 bar and 298 K, the LCD, the number of node connections (size), and the CO_2_ heat of adsorption (color). For the majority of MOFs with *Q*_st_ < 22 kJ/mol, CO_2_ adsorption capacity is very low and stays at <1 mol/kg. For the UiO family of MOFs, the amount of CO_2_ adsorbed decreases as the length of a linker—and therefore the pore size—increases from one benzene ring in UiO-66 to two and three benzene rings in UiO-67 and UiO-68, respectively. In all three structures, the CO_2_ uptake remains below 1 mol/kg. In general, the maximum CO_2_ uptake is obtained for structures with *Q*_st_ values >25 kJ/mol and LCD values of ca. 6–7 Å. The material with the highest predicted CO_2_ adsorption capacity is MOF-812^49^ with 2.5 mol/kg. The SBU in MOF-812 is connected to 12 tetrahedral linkers resulting in a 3D network of **ith** topology, which is different from the typical 12-connected Zr SBUs in the **fcu** series. To gain molecular-level insight into CO_2_ adsorption in MOF-812 and UiO-66, we compared their simulation snapshots at 0.15 bar and 298 K and found that CO_2_ molecules prefer to sit much closer to the Zr-nodes in MOF-812 (Figure 7a). This finding is also confirmed by the analysis of the radial distribution function (RDF) of atom pairs between the framework Zr and the oxygen atom in CO_2_ (Figure 7b). In MOF-812, the first peak appears at 2.5 Å, while for UiO-66, another structure with 12-connected nodes, we observe a much larger distance between Zr and CO_2_ molecules, with the first peak rising at 5 Å. Analysis of the pore size distribution (PSD) shows that MOF-812 contains smaller pores (4 and 5.5 Å) compared to those of UiO-66, whose small and large pores are 6.8 and 7.7 Å, respectively. The simulation snapshots show that in UiO-66, CO_2_ molecules reside in the small tetrahedral pores, whereas in MOF-812, CO_2_ molecules preferentially sit in the space between the Zr-oxide nodes in addition to the pockets in between the tetrahedral linkers: see the blue CO_2_ molecules in Figure 7a. The proximity of CO_2_ molecules to the Zr-oxide nodes in MOF-812 is also explained by the dominant MOF–CO_2_ electrostatic interactions (Figure S8). Using our online Wiz visualization platform,^50^ one can dynamically probe the effects of different textural properties on CO_2_ adsorption capacity for all the Zr-oxide MOFs studied here.

To probe the sensitivity of CO_2_ uptake predictions to the chemistry of Zr-oxide nodes with less than 12 connections, we took one of the 8-connected structures (BOSZEQ^51^) and removed the water and hydroxyl groups from the Zr-oxide node before running CO_2_ adsorption simulations (Figure S7). In other words, the newly constructed structure (Zr_6_O_4_(OH)_4_(RCO_2_)_8_) differs in its SBU chemistry compared to the staggered mixed node proton topology^39^ where water and hydroxyl groups are connected to Zr atoms (Zr_6_O_4_(OH)_4_(OH)_4_(OH_2_)_4_(RCO_2_)_8_). The CO_2_ adsorption predictions for these two seemingly similar structures are significantly different (Figure S6). At 0.15 bar and 298 K, the staggered protonated coordination gives substantially lower CO_2_ uptake (0.16 mol/kg) than the structure with no water or hydroxyl groups (5.3 mol/kg). Such a significant increase in the CO_2_ uptake prediction is driven by the enhanced MOF–CO_2_ electrostatic interactions (Figure S8) and reveals the importance of the Zr-oxide node chemistry when dealing with MOFs with site defects (e.g., Zr-oxide SBUs with fewer than 12 connections). This suggests that care must be taken when conducting adsorption simulations for these systems.

### 2.4 Augmented Reality (AR) Visualization of Gas Adsorption Sites

**Figure 8:**
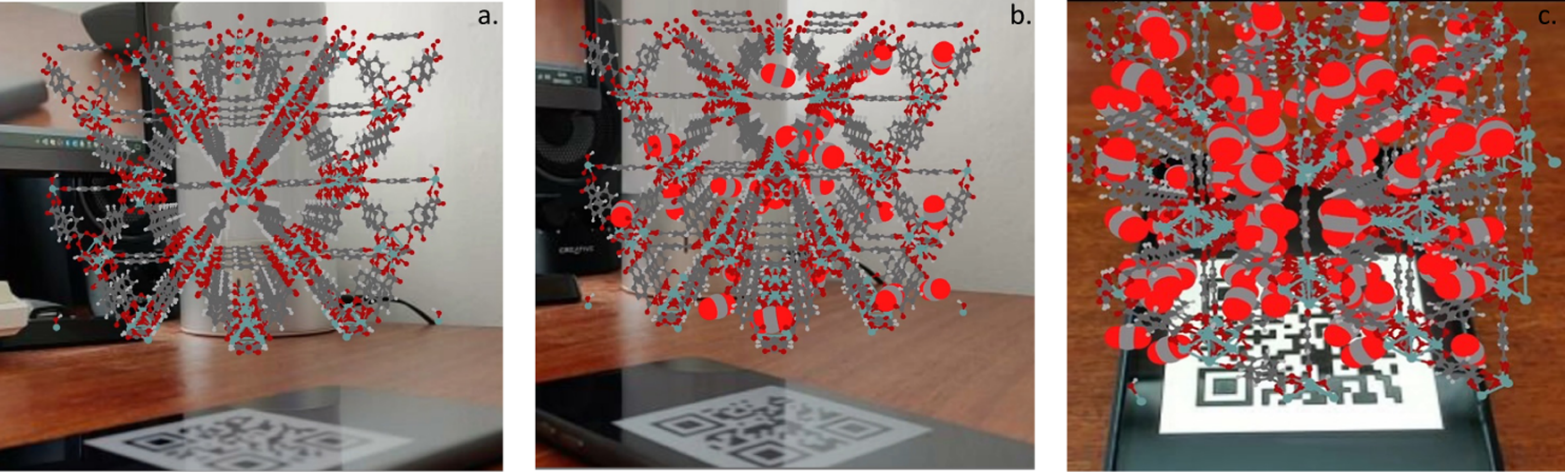
Augmented reality (AR) screenshots of (a) UiO-66 with CO_2_ molecules preferential adsorption sites at (b) at low pressure (1 bar) and (c) high pressure (10 bar). Detailed instructions for creating AR visualization of MOFs on mobile devices are provided in the Supporting Information (SI).

Provision of detailed information about MOF’s structural network, pores, surface chemistry, and preferential adsorption sites has been crucial in helping us to better understand the adsorption phenomena. Such information can be more useful and interactive if one can convey it in three-dimensional (3D) perspectives as more and more complex structures are designed and synthesized every day. A new emerging visualization technology that is widely used in other areas is augmented reality (AR). AR offers a seamless interface combining real and virtual worlds and has been employed as a teaching medium in the science field^52,53^ and used in the visualization of 3D molecular structures.^54^ In the present study, we use AR to not only visualize MOF structures but, more importantly, for the detection of favorable adsorption sites for gas molecules enabling experimental and computational MOF scientists to better understand the pore environment. To the best of our knowledge, this is the first AR visualization of gas adsorption in MOFs. Figure 8 demonstrates the application of AR for CO_2_ adsorption in UiO-66. GCMC simulation snapshots obtained at different CO_2_ loadings are used as input files for AR engine (Vuforia platform)^55,56^ to analyze CO_2_ adsorption sites at different loadings. At low pressures up to 1 bar (Figure 8b), CO_2_ molecules mainly occupy the tetrahedral cage and only start filling the octahedral cages when the pressure exceeds 10 bar. By creating different bar codes for each configuration, one can flick through different simulation snapshots and analyze the adsorption sites at different pressures (see the Video S1, Supporting Information). The AR visualization procedure, explained thoroughly in the methods section, can be applied to any MOF for detailed structural analysis and for the identification of adsorption sites in a more realistic way.

## 3 Methods

### 3.1 DFT Partial Atomic Charges Calculations

DFT simulations were performed with the fully periodic CRYSTAL17 software package.^57^ Simulations were initialized from previously reported single-crystal X-ray structures, which were obtained from the CCDC. Initially, five representative Zr-MOFs (BOGNES, BOSZEQ, MAFWEY, MUBZOA, and UiO-66) were selected for a comprehensive investigation into the influence of choice of density functional and basis set on the calculated partial charges. The five basis set were 6-31G(d,p), 6-31G(2d,2p), 6-311G(d,p), 6-311G(2d,2p), and def2-SVP. The five different functionals tested were PBE, PBE0, BLYP, B3LYP, and M06-2X. The convergence on the energy was set to Δ*E* ≤ 10-8 hartree, and a 2 × 2 × 2 Monkhorst–Pack grid was used to sample the reciprocal space lattice. Partial charges were calculated by subtracting the total atomic charge determined by the SCF electronic structure method from the atomic number. To evaluate if fully optimizing the geometries of the structures influenced the calculation of partial charges, we allowed the six structures to fully relax (atomic positions and lattice vectors) with an energy convergence criterion of Δ*E* ≤ 10^–8^ hartree. Ultimately, it was found that the differences in partial charges between the optimized and unoptimized structures were minimal, and thus, for the final simulations, the structures were used without optimization of the geometry. All of the chosen basis sets and potentials were then verified by comparing CO_2_ adsorption isotherms with published experimental data,^47,48^ focused on the UiO MOFs family as a benchmark.

### 3.2 Gas Adsorption Calculations

The adsorption isotherms were calculated using grand canonical Monte Carlo (GCMC) simulations. We used 5000 cycles for equilibration and 5000 cycles to average properties for each pressure point, where a cycle is defined as the maximum of 20 or the number of molecules in the system. The interactions between CO_2_–MOF and N_2_–MOF were modeled by Lennard-Jones (LJ) plus Coulomb potentials. LJ parameters for all atoms in MOFs were taken from the Dreiding force field^58^ except for the Zr atoms, for which we used the Universal force field (UFF) parameters. CO_2_ and N_2_ were modeled using the TraPPE force field.^59^ The details of the force field parameters are presented in Table S1 in the Supporting Information. The Lorentz–Berthelot mixing rules were employed to calculate fluid/solid LJ parameters, and LJ potential was cut-off at 12.8 Å. The Ewald summation technique was used to calculate all electrostatic interactions.

All frameworks were considered rigid during the simulations. Insertion, deletion, and translation moves were attempted in the simulations with equal probabilities. All of the simulations were carried out using the RASPA molecular simulation software^60^ and validated by comparison with published experimental data.

### 3.3 5D Visualization Platform and Data Analytics

All data sets plotted here can be reproduced online using the Wiz visualization dashboard:^50^ step-by-step instructions are provided at https://wiz.shef.ac.uk. Adsorption data as well as geometric properties are provided in the Supporting Information in a.csv file, which can be directly uploaded to Wiz for data visualization and analysis. Visitors to Wiz can generate structure–property relationships interactively by plotting variables into each of the five axes according to their interests. Users can also explore and search for MOFs by their name and geometric/adsorptive properties.

### 3.4 Augmented Reality Visualization

For AR visualization, two software packages (Jmol^61^ and Unity^62^) and the Vuforia platform (Augmented Reality engine)^55^ were used. Jmol was used to convert molecular structure files (.mol and.cif) into object files (.obj and.mtl), which can be imported to Unity. Unity was then used to set up the application and the Vuforia AR Engine and to assign the molecule objects to specific target images, which are set up through a Vuforia’s online platform. A step-by-step guide for AR visualization of MOFs has been provided in the Supporting Information (Section S8) along with the supported screenshots and video link.

## 4 Conclusions

In this work, we generated a subset of 102 Zr-oxide MOFs deposited in the Cambridge Structural Database. For all structures, we characterized full geometric properties and successfully assigned their topologies. We also simulated full N_2_ adsorption isotherms and calculated the surface area by rigorously applying the consistency criteria from the BET theory. When compared with geometrically calculated accessible surface area (ASA), in general, BET area calculations are lower than ASA for most Zr-oxide MOFs, especially those with micropores, as well as structures with the **csq** topology. The BET area characterization performed here offers experimentalists a benchmark for the quality assessment of the Zr-oxide MOFs: ensuring the BET consistency criteria are met, we suggest that the experimental BET area is compared with the simulated BET area and not ASA.

We also calculated and assigned partial atomic charges for 37 Zr-oxide MOFs using systematic periodic DFT calculations comparing 25 different combinations of basis sets and functionals. We validated the DFT calculations by comparing simulated CO_2_ adsorption isotherms with published experimental data for UiO-66 and UiO-67. We found that the combination of PBE0 functional with the def2-svp basis set provides an excellent match with the experimental data. We then performed high-throughput GCMC simulations and derived structure–property relationships for CO_2_ adsorption in Zr-oxide MOFs. We found that the maximum CO_2_ uptake is obtained for structures with the heat of adsorption values >25 kJ/mol and the largest cavity diameters of ca. 6–7 Å. Importantly, we found that slight changes in the chemistry of Zr-oxide nodes result in significant changes in CO_2_ adsorption predictions. For example, in BOSZEQ, when the proton topology of staggered water/hydroxyl is present on the Zr-node, substantially lower CO_2_ uptake is predicted (0.16 mol/kg) compared to the case where water and hydroxyl groups are removed (5.3 mol/kg). Therefore, when comparing adsorption simulations and experiments, care must be taken when defining the chemistry of defective Zr-MOFs. All data presented in this paper can be uploaded and reproduced in Wiz, our web-based data analytics/visualization platform, allowing users to interactively explore structure–property correlations and search for structures with optimal structural and adsorptive properties.

In addition, we applied augmented reality (AR) to not only visualize and understand the complexity of Zr-oxide nodes but also introduced it as an educational tool to bring alive adsorption phenomena in porous materials, revealing both favorable adsorption sites and gas capture interactions. It is our hope that such tools will ultimately augment material scientists’ expertise to design functional materials using atomic- and molecular-level insights.

## Supporting Information Available

Augmented Reality visualization video (MP4)
Spreadsheets for geometric properties and topology characterization (XLSX)
Spreadsheets for CO_2_ adsorption calculations (XLSX)
Spreadsheets for N_2_ adsorption and BET area calculations (XLSX)
Spreadsheets for PSD calculations (XLSX)
Crystallographic data (ZIP)
Search criteria for Zr-oxide MOFs in ConQuest, adsorption isotherms and AR visualization (PDF)
