# Supplementary material for: Computational Characterization of Zr-Oxide MOFs for Adsorption Applications
Source: ACS Appl Mater Interfaces. 2022 Dec 14;14(51):56938–47. doi: 10.1021/acsami.2c13391 (PMC9801377; doi:10.1021/acsami.2c13391)
Supplement: Supplementary file 7 — am2c13391_si_007.pdf [file am2c13391_si_007.pdf]

Supporting Information

Computational Characterisation of Zr-oxide MOFs for Adsorption Applications

Rama Oktavian,[a] Raymond Schireman,[c] Lawson T. Glasby,[a] Guanming Huang,[a] Federica Zanca,[a] David Fairen-Jimenez,[d] Michael T. Ruggiero[c] and Peyman Z. Moghadam *[a,b]

[a]Department of Chemical and Biological Engineering, The University of Sheffield, Sheffield S1 3JD, UK

[b]Department of Chemical Engineering, University College London, London WC1E 7JE, UK

[c]Department of Chemistry, The University of Vermont, Burlington, VT 05405, USA

[d]Department of Chemical Engineering & Biotechnology, University of Cambridge, Philippa Fawcett Drive, Cambridge CB3 0AS, UK

Correspondence and requests for materials should be addressed to Peyman Z. Moghadam (p.moghadam@ucl.ac.uk)

S1. Search criteria in CCDC's structure search software, ConQuest

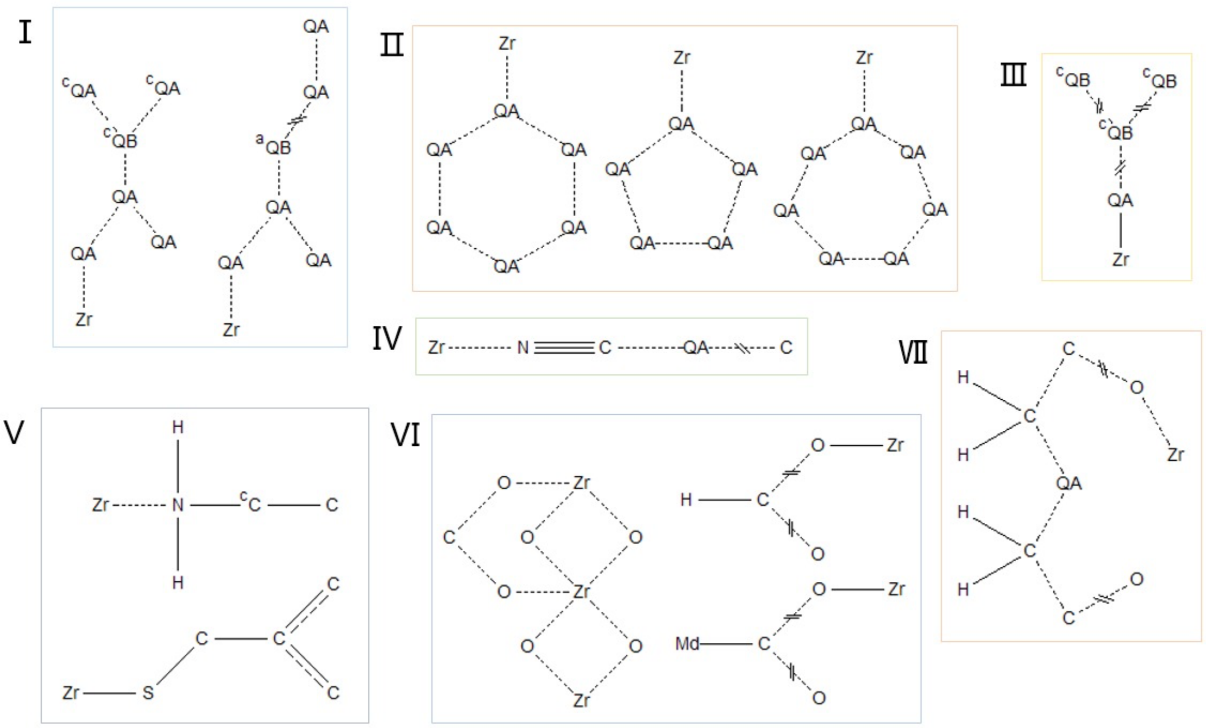

Figure S1. Seven criteria developed for searching for Zr-oxide MOFs in the CSD MOF subset. QA = O, N, P, C, B, S. QB = N, P, B, S, C and superscripts "c" and "a" impose the corresponding atoms to be "cyclic" or "acyclic", respectively. Me denotes methyl groups (redrawn from the work of Moghadam et al.[1]).

S2. Constructing hydroxylated Zr-oxide clusters

Figure S2 shows UiO-66, a well-known Zr-oxide MOF that have been reported in two forms: hydroxylated ($Zr_6O_4(OH)_4$) and dehydroxylated (Zr_6O_8).[2] For consistency across all Zr-MOFs, before running the periodic DFT calculations, we manually added the hydroxyl groups for every structure extracted from the CSD MOF subset.

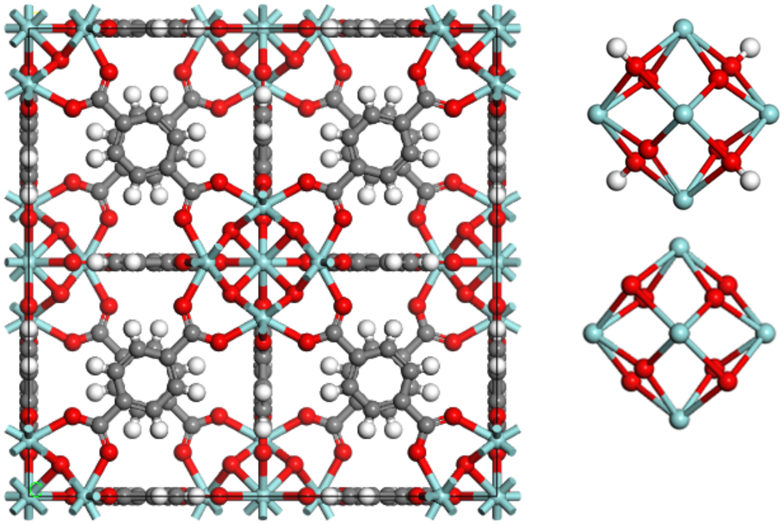

b

c

a

Figure S2. a. The UiO-66 structure. Grey: C, white: H, red: O, cyan: Zr. **b.** hydroxylated and **c.** dehydroxylated Zr-oxide node.

S3. N_2 adsorption isotherms and BET area calculations

Nitrogen adsorption isotherms (77 K) in all studied Zr-oxide MOFs were calculated via grand canonical Monte Carlo (GCMC) simulations using RASPA simulation software.[3] 10^4 Monte Carlo cycles were performed, the first 50% of cycles were applied for equilibration, and the remaining cycles were applied to calculate the ensemble averages. Insertion, deletion, rotation, and translation moves were set at equal probability. The framework atoms were kept fixed at the crystallographic positions for all Zr-oxide MOFs. Adsorbate-adsorbate and adsorbate-adsorbent interactions were modelled using a Lennard-Jones (LJ) plus Coulomb potential. The force field parameters for nitrogen and carbon dioxide were taken from the TraPPE force field. All force field parameters are tabulated in Tables S1-S3.

Table S1. LJ parameters for the Zr-oxide MOFs.

Atom type	σ (Å)	ε/k_B (K)	Force field
**C**	3.473	47.856	Dreiding[4]
**O**	3.033	48.158	Dreiding[4]
**H**	2.846	7.649	Dreiding[4]
**N**	3.263	38.949	Dreiding[4]
**Zr**	2.783	34.722	UFF[5]

Table S2. LJ parameters and partial charges for N_2.

Atom type	σ (Å)	ε/k_B (K)	Atomic charge	Force field
**N_N_2**	3.31	36.0	-0.482	TraPPE[6]
**N_center of mass**	0	0	0.964	TraPPE[6]
**N_N_2**	3.31	36.0	-0.482	TraPPE[6]

Table S3. LJ parameters and charges for CO_2.

Atom type	σ (Å)	ε/k_B (K)	Atomic charge	Force field
**C_CO_2**	2.80	27.0	0.70	TraPPE[6]
**O_CO_2**	3.05	79.0	-0.35	TraPPE[6]

The procedure for BET area calculations is explained in detail as follows:

1) According to the BET theory[7], monolayers usually form at pressures of $0.05 < P/P_0 < 0.3$. We chose this range as the first guess.
2) Plot the left side of eq. (1) versus selected range of relative pressure, perform linear regression to obtain values for C and Nm.

$$\frac{P/P_0}{N(1-P/P_0)} = \frac{1}{N_m C} + \frac{C-1}{N_m C}\left(\frac{P}{P_0}\right) \qquad (1)$$

3) Check compliance with consistency criteria 1 and 2.
4) Check whether the selected range from step 1 satisfy criteria 3. If not, pick another range of relative pressure and start from step 2 again.
5) Calculate the value of $(1/\sqrt{C} + 1)$. Check whether the selected range from step 1 satisfies criteria 4.[8] If not, pick another range of relative pressure and start from step 2.
6) Calculate the BET area using eq. 2.

$$S = N_m . A_{N_2} . N_{AV} . \hat{V}_{N_2} \qquad (2)$$

where: S = surface area, N_m = nitrogen monolayer uptake in m^3(STP)/g, A_{N2} = cross section of nitrogen molecule (1.62×10^{-19} m^2/molecule), N_{AV} = Avogadro number (6.022×10^{23}), $\hat{V}_{N_2}$ = nitrogen molar volume at STP (44.64 mol/m^3)

Figure S3 shows an example of N_2 adsorption isotherm in MOF-812 followed by BET area calculations. We note that we have provided N_2 adsorption isotherms and BET area calculations for all 102 Zr-oxide MOFs in the supporting information.

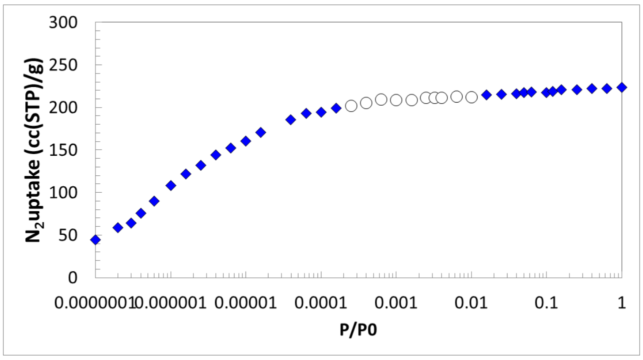

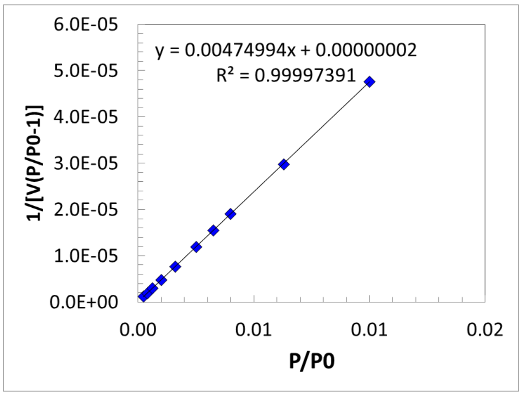

	range 1								
slope	0.00474994								
intercept	0.00000002								
C=1+slope/intercept	237498	0.002047763							
A=1/(slope+intercept)	210.5280887								
uptake monolayer cm3(STP)/g	210.53								
cross section (m2/molecule)	1.62E-19								
molar volume (STP) (mol/m3)	44.64117195								
uptake monolayer m3(STP)/g	0.000210528								
avogadro (Nav)	6.02E+23								
BET surface area (m2/g)	917								
P/Po range	BET SA	A	R	N2@ upper P/Po	N2@lower P/Po	1/(√C+1)	C	P(A) read	%e P(A)
	m2/g	cc(STP)/g		cc(STP)/g	cc(STP)/g				
0.0003-0.0100	917	210.53	0.999974	212.02	201.79	0.00205	237498.0000	0.0022926	-10.68

Figure S3. Example BET area calculation for MOF-812 (CCDC refcode: BOHWOM). Left panel shows the calculated nitrogen adsorption isotherm: white symbols indicate the pressure range used for BET area calculation. Right panel shows the BET plot used for the first consistency criteria. Bottom panel presents the calculations used to test compliance with all four consistency criteria suggested by Rouquerol *et al.*[9]

The four consistency criteria proposed by Rouquerol *et al.*[9] are described in details here:

1) Only a range where $N(1 - P/P_o)$ increases monotonically with P/P_o should be selected.
2) The value of C resulting from the linear regression should be positive.
3) The monolayer loading N_m should correspond to a relative pressure P/P_o falling within the selected linear region.
4) The relative pressure corresponding to the monolayer loading calculated from BET theory $(1/\sqrt{C} + 1)$ should be equal to the pressure determined in criterion 3. (For this criterion, Rouquerol *et al.*[9] suggested a tolerance of 20%.)

S4. CO_2 adsorption isotherms in UiO-66 and UiO-67.

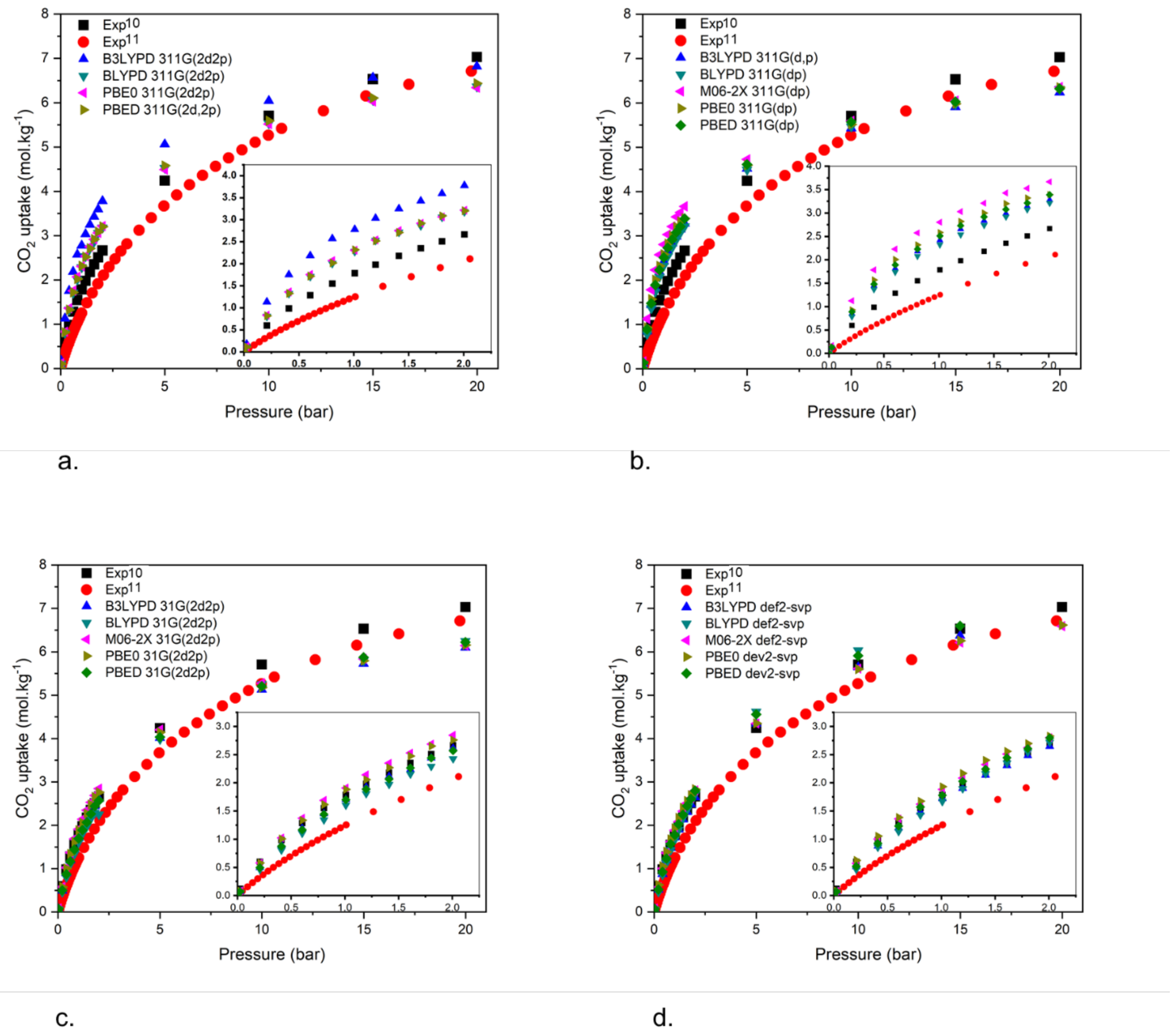

Figure S4. CO_2 adsorption isotherms in UiO-66 at 298 K. Each simulated isotherm is obtained from a different combination of basis set and functional: **a.** 6-311G(2d,2p); **b.** 6-311G(d,p); **c.** 6-31G(2d,2p); **d.** dev2-svp. Experimental isotherms are shown for comparison.[10,11]

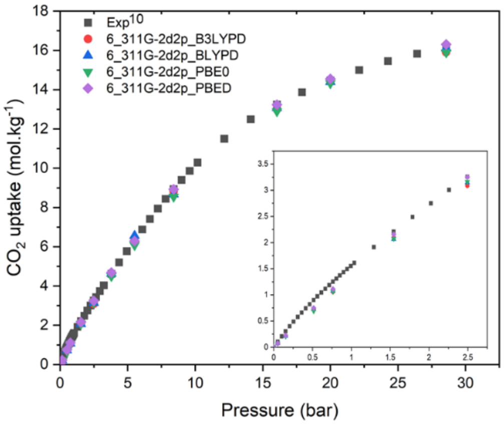

a.

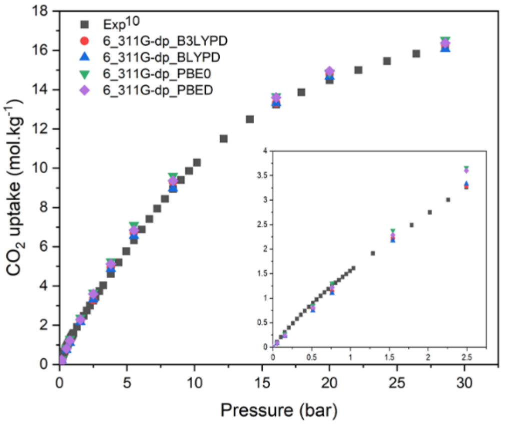

b.

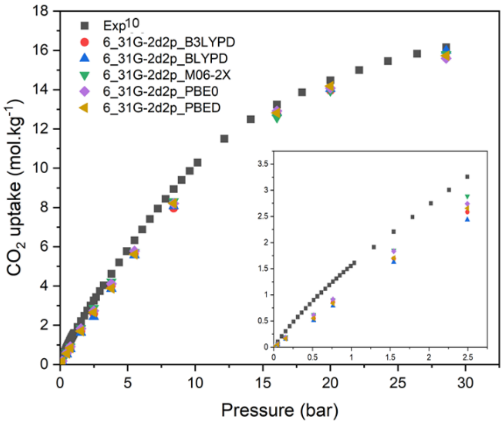

c.

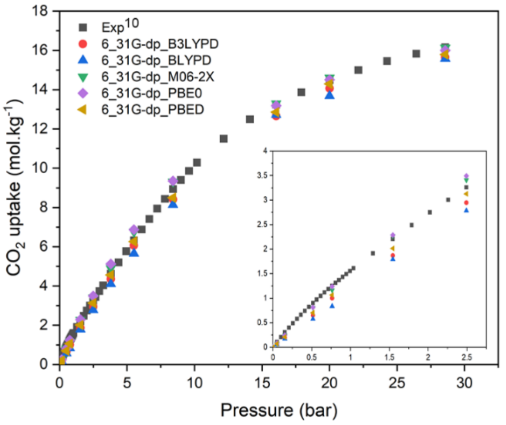

d.

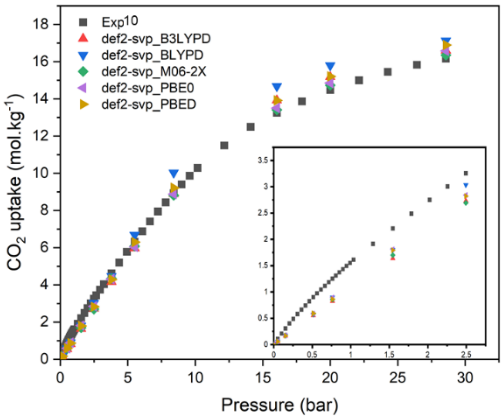

e.

Figure S5. CO_2 adsorption isotherms in UiO-67 at 298 K. Each simulated isotherm is obtained from a different combination of basis set and functional: **a.** 6-311G(2d,2p); **b.** 6-311G(d,p); **c.** 6-31G(2d,2p); **d.** 6-31G(d,p); **e.** dev2-svp. Experimental isotherms are shown for comparison.[10]

S5. CO_2 adsorption isotherms for BOSZEQ structures.

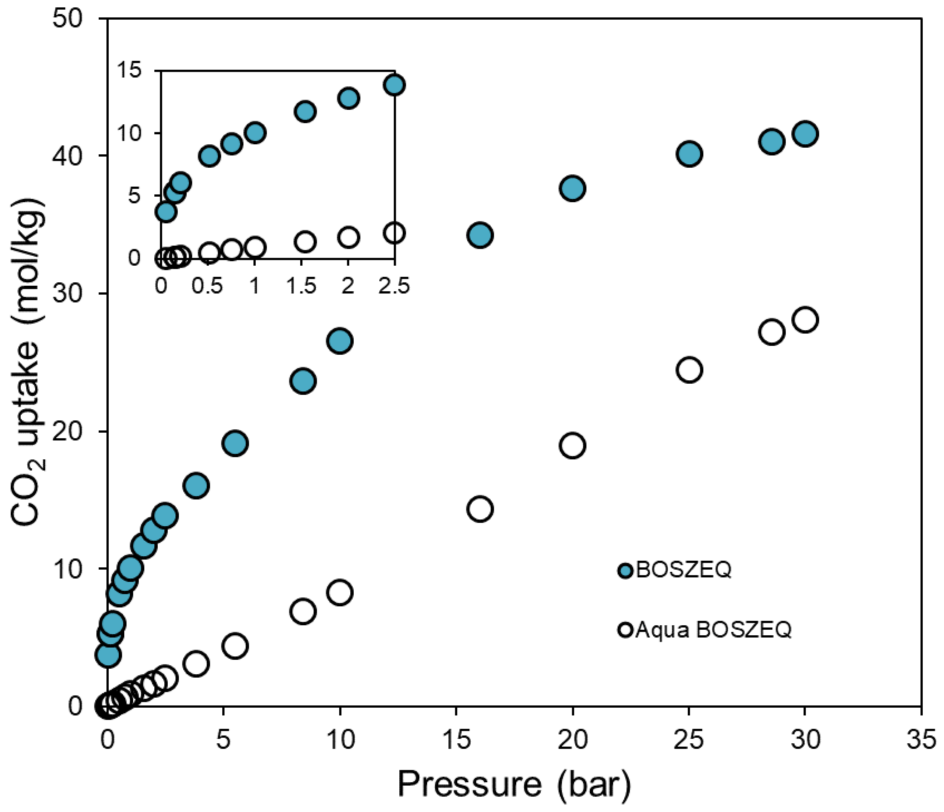

Figure S6. CO_2 adsorption predictions in BOSZEQ (no water) and aqua BOSZEQ at 298 K, inset shows CO_2 adsorption at low pressure regime. The aqua BOXZEQ structure contains the staggered mixed node proton topology.

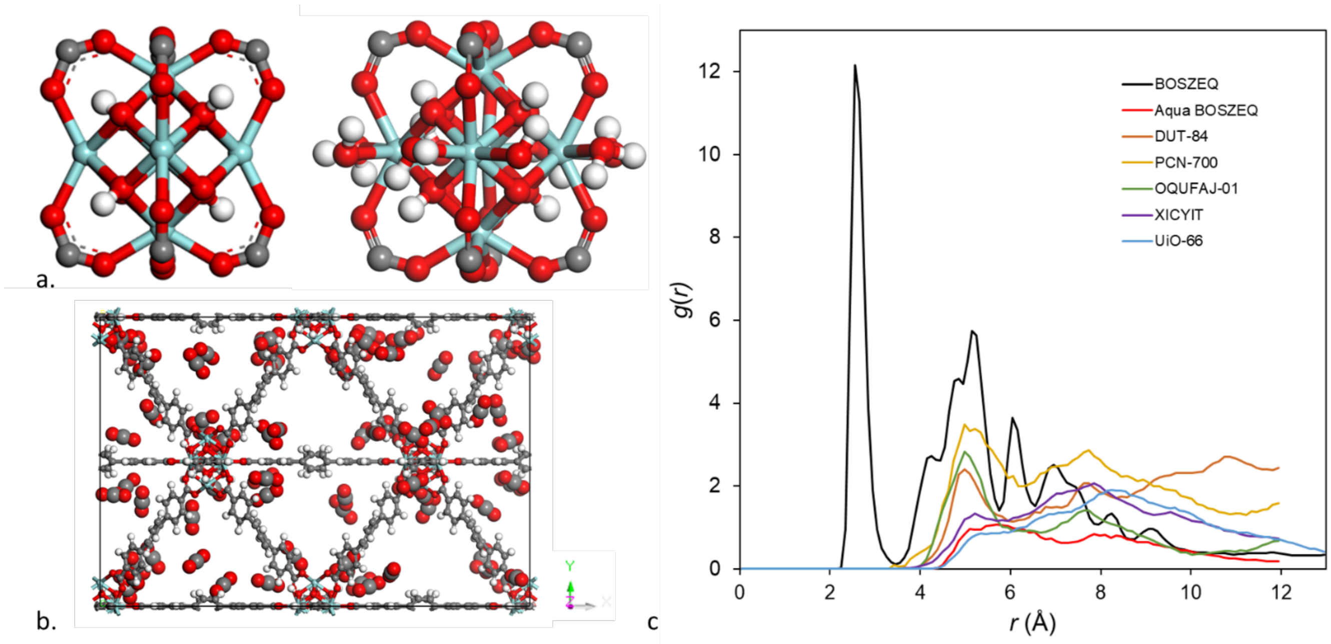

Figure S7. a. Metal cluster in BOSZEQ and BOXZEQ structure with the staggered mixed node proton topology i.e. aqua BOSZEQ; **b.** CO_2 adsorption snapshot in BOSZEQ simulated at 0.15 bar and 298 K; **c.** Radial distribution functions between Zr of the metal node and O of CO_2 molecules for selected MOFs. For aqua BOSZEQ, the presence of water molecules prevents CO_2 molecules sitting close to the pockets in between ligands and therefore the first RDF peak appears at ca. 5 Å. The proximity of CO_2 molecules to the Zr-oxide nodes in BOSZEQ is explained by the dominant MOF-CO_2 electrostatic interactions.

S6. MOF-CO_2 electrostatic interactions.

DFT simulations were performed with the fully periodic CRYSTAL17 software package.[12] Framework Partial charges were calculated by subtracting the total atomic charge determined by the SCF electronic structure method from the atomic number. For BOSZEQ, EMIYUW, OFAWID, OQUFAJ01-03, QOKBOJ, RUBLAD, UNEJEE, XICYIT and DITJOH structures, we used PBE0 functional and DDEC[13] charge partitioning approach.

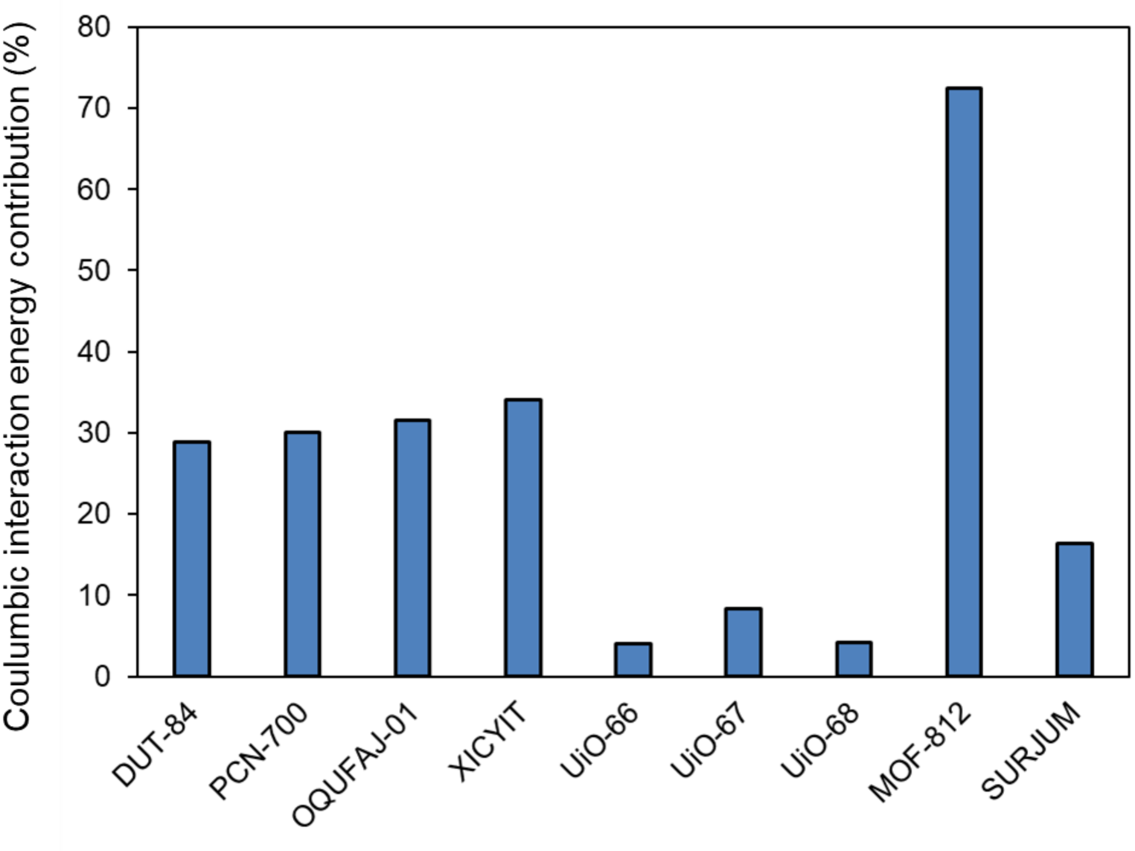

Figure S8. The comparison between MOF-CO_2 Coulombic interaction energy for selected MOFs at 0.15 bar and 298 K.

S7. Structural properties of top-performing Zr-MOFs for CO_2 capture.

MOF-812

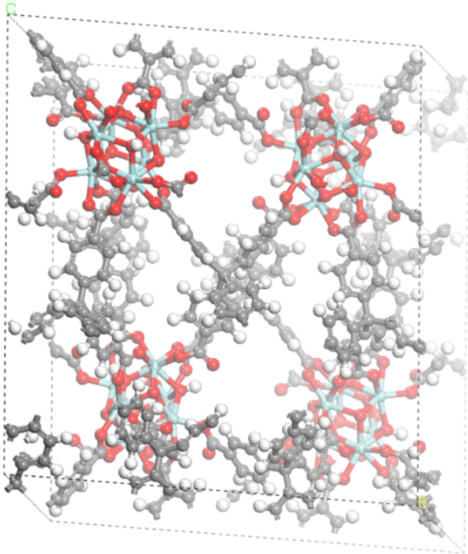

CCDC ref code: BOHWOM
LCD: 6.0 Å
PLD: 4.6 Å
Density: 1.183 g/cm^3
Void fraction: 0.49
Number of connected ligands: 12
Topology: *ith*
CO_2 uptake: 2.50 mol/kg

SURJUM

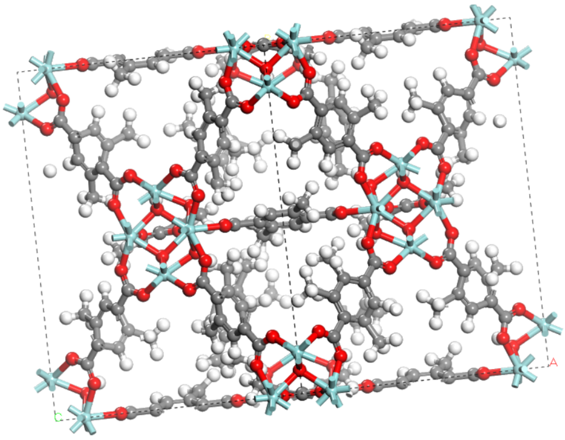

CCDC ref code: SURJUM
LCD: 6.7 Å
PLD: 2.8 Å
Density: 1.352 g/cm^3
Void fraction: 0.44
Number of connected ligands: 12
Topology: *fcu*
CO_2 uptake: 1.98 mol/kg

XICYIT

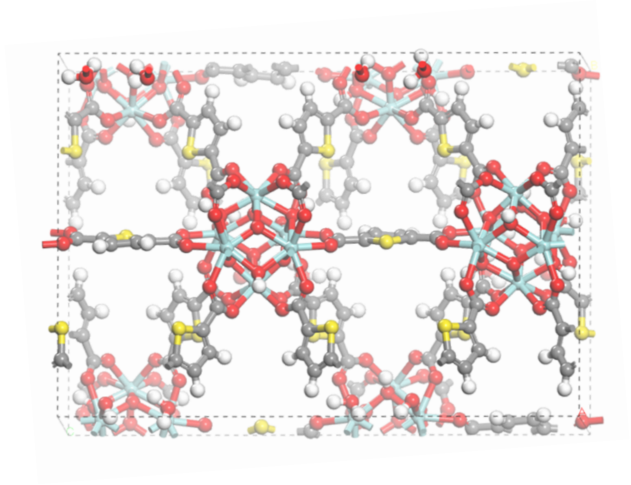

CCDC ref code: XICYIT
LCD: 6.0 Å
PLD: 3.4 Å
Density: 1.332 g/cm^3
Void fraction: 0.57
Number of connected ligands: 8
Topology: *bct*
CO_2 uptake: 1.98 mol/kg

S8. Metal Organic Frameworks (MOFs) structure visualization using Augmented Reality (AR).

Instruction on how to visualize the structure of MOFs using AR

Porous materials such as MOFs has been emerging as new class materials for severalapplications in chemical industries such as gas adsorption and separation, catalysis, and energy storage material. As of January 2020, it is highly noted that there are 99,075 MOFs available in the Cambridge Structural Database (CSD) MOFs subset.[14] The information about the structure of this materials is essential to be provided and more interestingly if this information could be displayed in three-dimensional (3D) perspectives. However, it may be difficult to convey that information in two-dimensional (2D) space such as paper or computer. Augmented Reality (AR) could help to address this issue where this technology has been being intensively used in computer games and films but underused in chemical science field. This instruction will give you the step by step how to visualize the 3D structure of MOFs in 2D spaces according to the previous work.[15] It is expected that this instruction could become tools in teaching of chemical science and help the students to understand about geometric structures of porous materials, i.e MOFs.

The outline of the instructions explained here has been taken from previous work.[15] Two software (Jmol[16] and Unity[17]) and the Vuforia platform[18] (Augmented Reality engine) are needed for the MOFs visualization. All of these programs are free to use. Jmol is used to convert molecule structures files (.mol and .cif) into object files (.obj and .mtl) which can be imported to Unity. Unity is used to setup the application and the Vuforia AR Engine and to assign the molecule objects to specific target images which are setup through Vuforia's online platform. If desired, Unity is also able to buildthe project into an APK file which can be installed as an app on Android phones. Applications designed by Unity can also be published onto the Play Store and App Store for Apple devices.

Below are the links for downloading all of those software:

- Download Jmol here: http://jmol.sourceforge.net/download/
- Jmol is a Java based application which requires Java to be installed. Java isavailable here: https://java.com/en/download/Unity (Individual License) : https://unity3d.com/get-unity/download
- When installing Unity, ensure the **Android/iPhone Development Packages** are selected if you are interested in viewing the AR molecules on your phone.
- Mercury is an optional program to view and manipulate the MOL and CIF files.It is however **required** if you want to show packing structures of MOFs: https://www.ccdc.cam.ac.uk/Community/csd-community/freemercury/

Here are the step by step process on how to visualise MOFs structure:

1. Molecule Structure files

- Obtaining MOFs structure files

a. Unity accepts molecular models as Object files (.obj & .mtl) which can bemodelled yourself using a program like Blender. Alternatively, the .mol and .cif files which already exist on the CSD MOFs subset[19] can be converted into these Object files using Jmol.

b. If you require the MOF packing to be viewed rather than the individual MOF molecule, then the CIF file should be downloaded from the Cambridge Crystallographic Data Centre (CCDC) rather than the MOL file.

c. To download a MOL file, search the CCDC database (https://www.ccdc.cam.ac.uk/structures/) for the desired MOF and on theviewer, select "Open" and "CSD Entry in External Viewer" shown in Fig. S9.

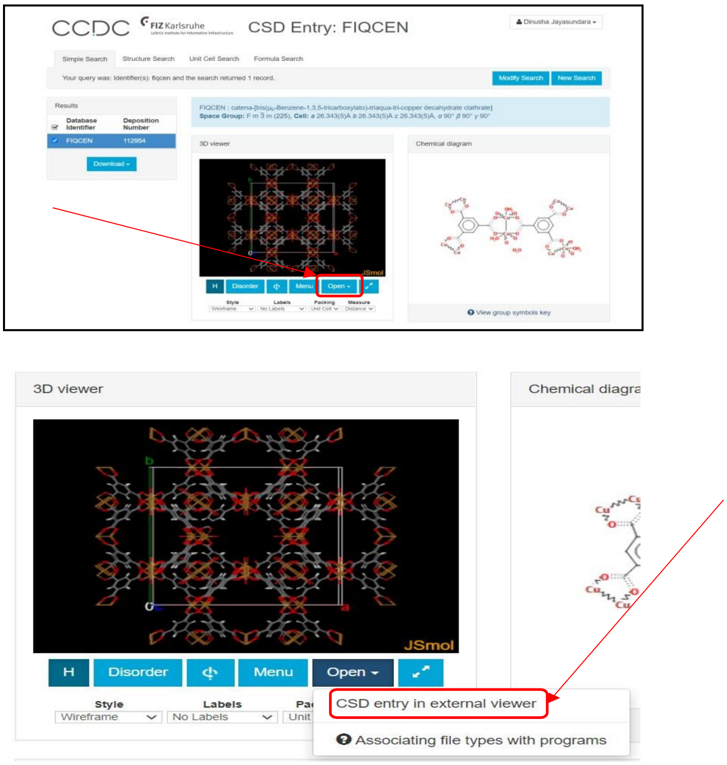

Figure S9. The CCDC database web page display

- Packing Models

a. By default, the MOL and CIF files downloaded from the CCDC will only show the single MOF molecule. If the packing model of the MOF needs to be shown instead, the CIF file and Mercury will be required.

b. To show the MOF in its packing structure, open the CIF file in mercury, right click, go to "Packing" and select "Packing". This can alternatively be done from the bottom window displayed in Fig. S10.

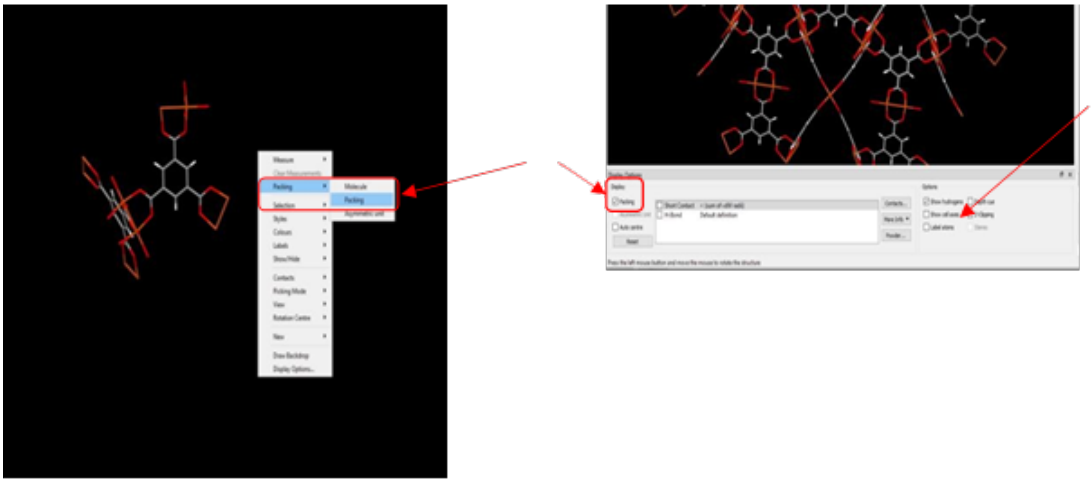

Figure S10. The packing model in Mercury software

c. Disable the axes from the bottom window and save the file as a “Mol2” file (allows for larger structures with more atoms)

2. Converting Molecule Structure to Object Files

• Jmol: Converting into Object Files

a. Now you have a mol file or mol2 file (for MOFs shown in packing structures), they can be converted into object files suitable for Unity.

b. Once Jmol has been installed, use the jmol.bat file in the install folder to start the program. If there are any problems loading the program, ensure you have Java installed. Jmol can convert mol, mol2 and CIF files into object files.

c. Right click and go to the “Style” submenu and ensure Axes, “Boundbox” and “Unit cell” are set to “Hidden” to ensure these elements aren’t added into the model when the file is converted into an object. Under the Style submenu, the scheme of the models can also be changed for example from ball and sticks to wireframe shown in Fig. S.11. I find sticks works best for large molecules.

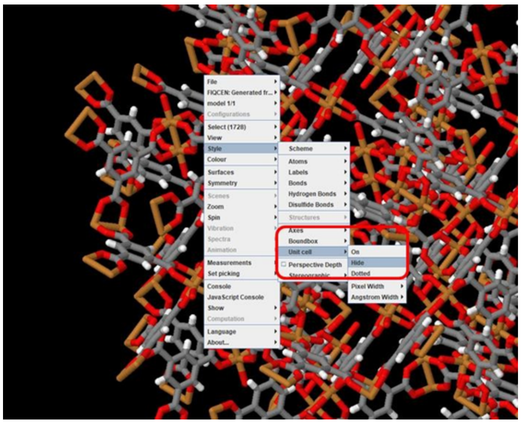

Figure S.11. Conversion of molecule structure to object files in Jmol

d. Alternatively go to File -> Open Console and enter “unitcell off; boundbox off; axes off” as displayed in Fig. S.12.

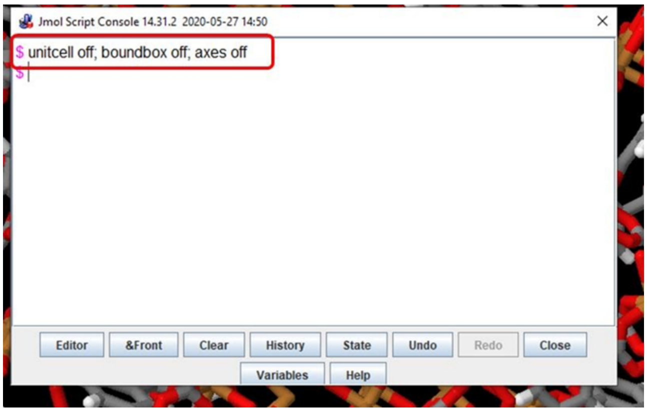

Figure S.12. Jmol Script Console display

e. To convert the MOL/CIF file in to an object, go to File and open the console.

f. Enter “write filename.obj” into the console where filename is a name of your choosing.

g. Once that command has replied with an “OK” output, the .obj and .mtl files are ready for use. These files are by default saved in the same folder as Jmol (Fig. S.13).

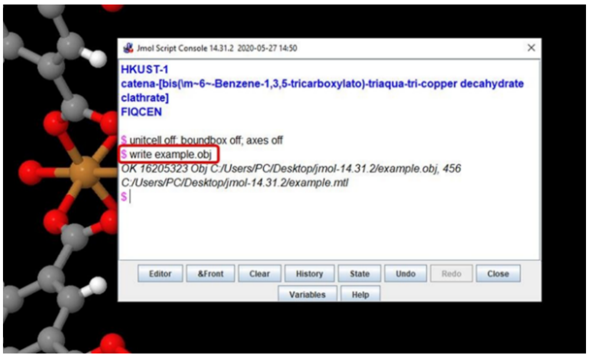

Figure S.13. Conversion of molecule structure to object files in Jmol Script Console

3. Setting Up Vuforia

- Setting up License

a. This is the augmented reality engine that works with unity and has a website which allows you to set up target images for the app.

b. Create an account by registering through this website https://developer.vuforia.com/vui/auth/register. Once registered, log in and go to “License Manager” and “Get a Development Key” (Fig. S.14).

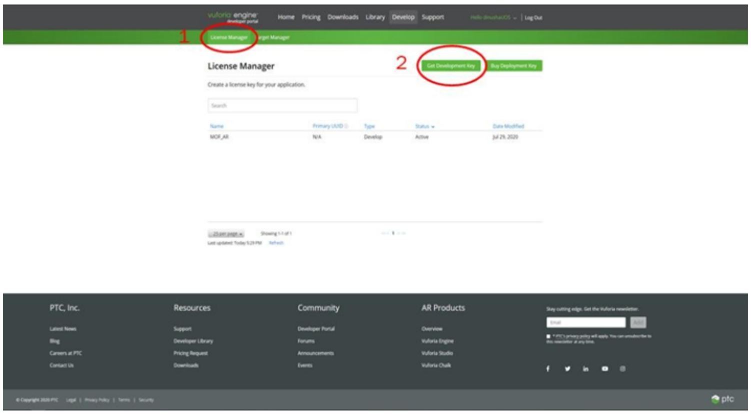

Figure S.14. Vuforia license manager web page display

c. Name this license what you like, however note that it has limited use as it is a free license (Fig. S.15).

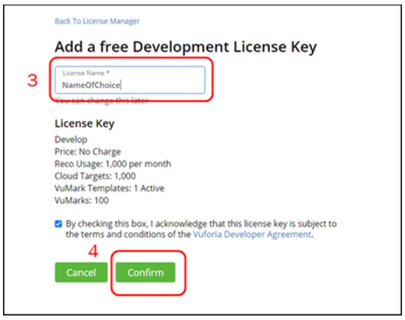

Figure S.15. Vuforia license key display

d. Once this license is made, click on it from the "License Manager" screen and copy and make a note of the following License Key which will be required later in Unity (Fig. S.16). The license is now setup and we can move on to the next stage in setting up Vuforia which is assigning a Target Image.

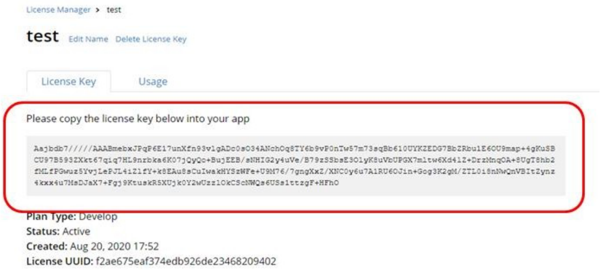

Figure S.16. License key

- Targeting Image

a. The target image can be any .jpg image of your choice. For this example, a 2D representation of a MOF from the CSD has been chosen. The higher the contrasting colours and shapes within an image, the better a target it will be and will result in better tracking and quicker projection when viewing in AR.

b. Download a target image of your choosing and go to “Target Manager” and create a new “Device” type database to store your target images under (Fig. S.17 (a) and b). Once the database is made, select it from the Target Manager section to begin adding the desired Target Images.

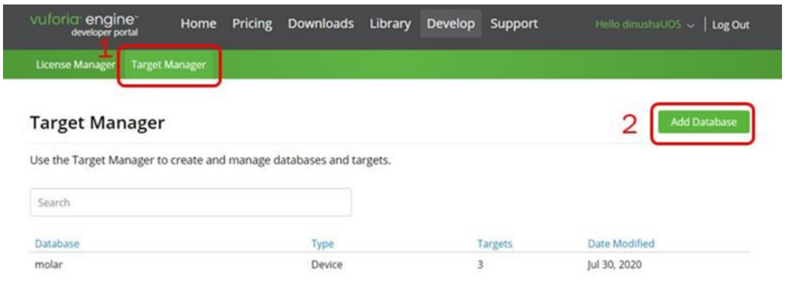

(a)

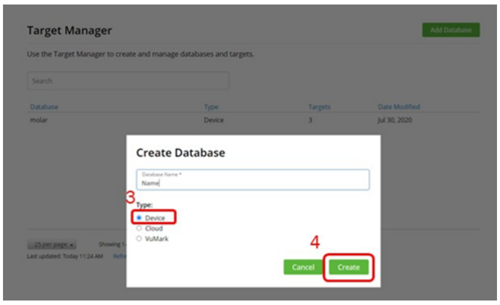

(b)

Figure S.17. Target manager page display

c. Go to “Add Target” and upload your desired target image (Fig. S.18 (a)). Jpeg formats seem to be accepted most consistently (programs like IrfanView or Paint can be used to ensure your image file formats and size meet the requirements).

d. Select the desired image and enter a desired value for width (default of 10 seems to work for use with MOF models) (Fig. S.18 (b)). This only defines the default image size in Unity which can be scaled up or down later. Add as many target images as you would like to. For simple applications, having one target image for each MOF you would like to present in AR works best.

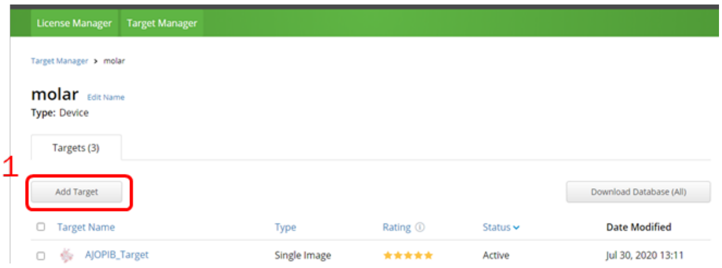

(a)

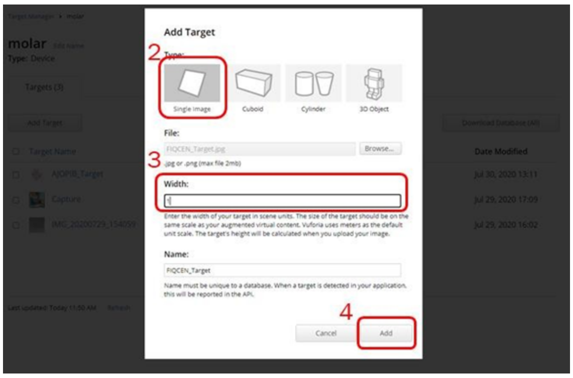

(b)

Figure S.18. The page for adding the image target

e. Once all the desired target images have been added, go to "Download Database" (Fig. S.19 (a)) and download it for "Unity Editor" (Fig. S.19 (b)). This will download a Unity package which can be used later to add these target images into the Unity app in a way which the Vuforia plugin will recognise.

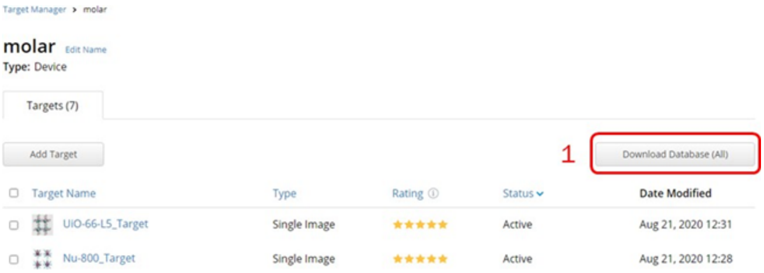

(a)

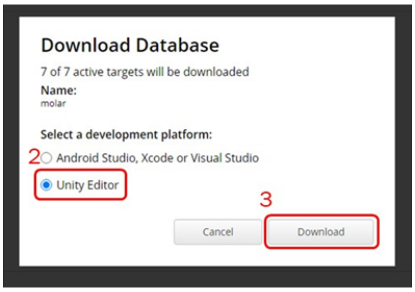

(b)

Figure S.19. Target image for Unity editor

f. The final thing to do on the Vuforia website is to download the Vuforia unity plugin which is used to add the Vuforia engine into your Unity Project. This is found under the Downloads section of the Vuforia webpage (Fig. S.20).

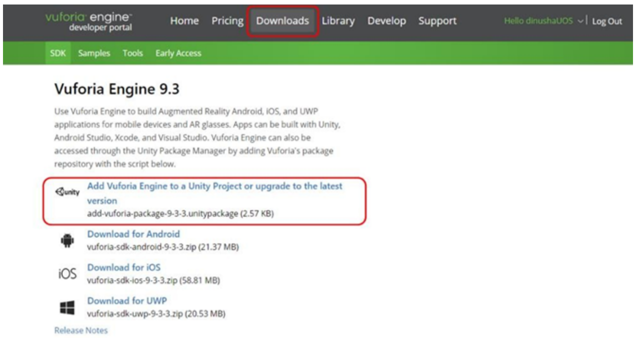

Figure S.20. Vuforia plugin download website page for Unity editor

4. Using Unity

- Setting up Unity

a. Open Unity and start a new project (Fig. S.21 (a))

b. Select 3D Template and choose a name and location for your project (Fig. S.21 (b))

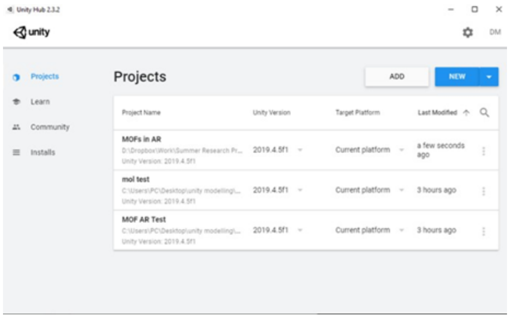

(a)

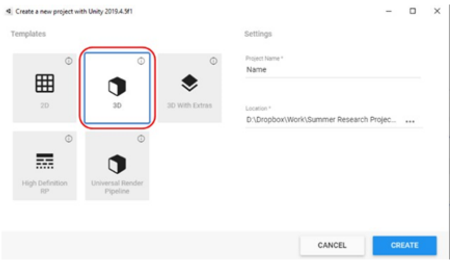

(b)

Figure S.21. Unity interface and setting up process

- Importing MOF object

a. Once the project is open, go down to the Assets folder and create a new folder in it called molecules.

b. Drag and drop each molecule MTL file followed by the corresponding OBJ file (Fig. S.22). Note, for larger molecules it can take some time for the OBJ files to be processed. Additional MOF MTL & OBJ files should be added to this folder.

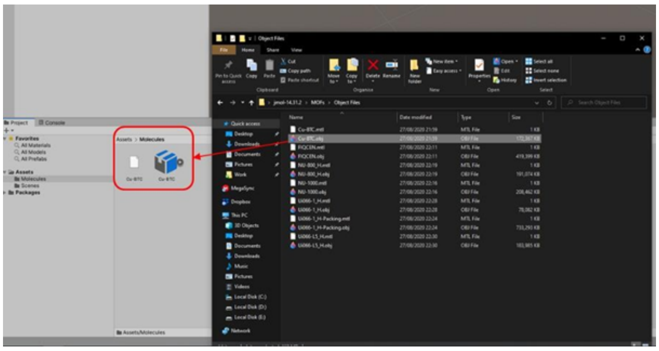

Figure S.22. Importing .obj file to Unity

c. Once loaded, the cube OBJ files in this folder can be dragged into the main scene window.

- The Vuforia plugin installation

a. For the AR part of the project, the Vuforia Plugin Package that was downloaded earlier should be added to the project.

b. Find the “add-Vuforia…” unity package file in your downloads and drag and drop it into Unity.

c. Press Import on the Package Import window to add the Vuforia Package to Unity (Fig. S.23). If any errors appear on the bottom left corner of the screen, exit and reload the project. They are usually not persistent.

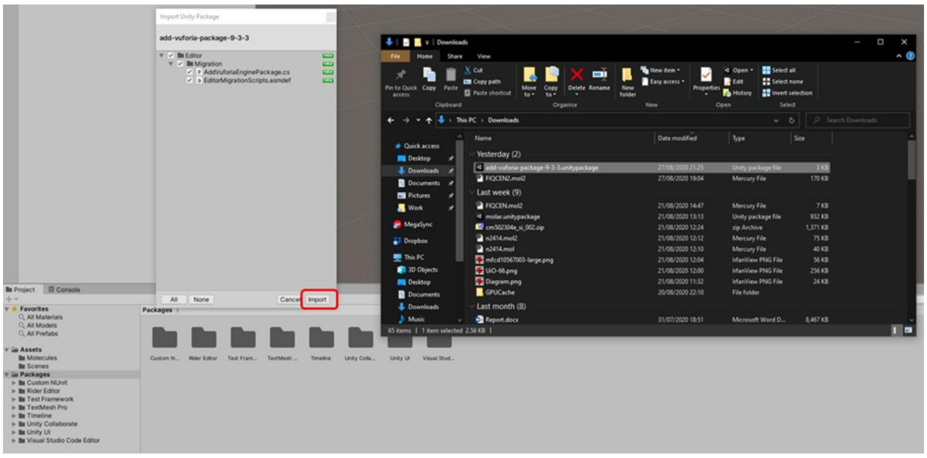

Figure S.23. Vuforia plugin installation step for Unity

- Adding target image database

a. Open the target image database Unity Package file that was downloaded from Vuforia’s database page.

b. Click Import in the new window which pops up to get this target image database into your project ashown in Fig. S.24.

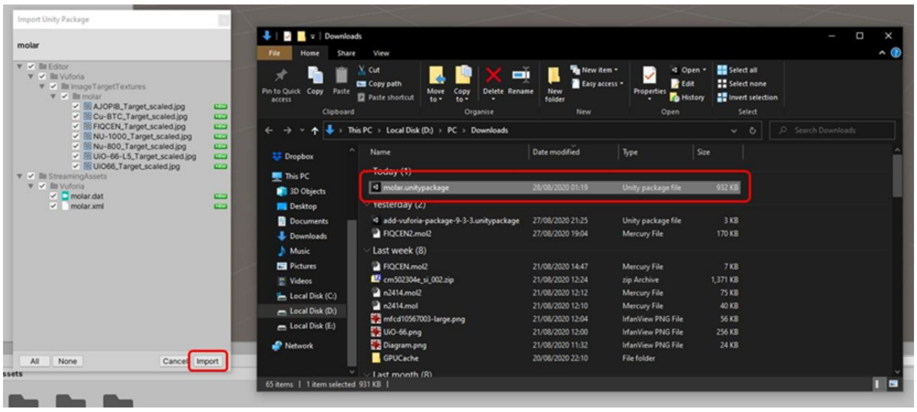

Figure S.24. Adding target image step

- Setting up the scene

a. In the Hierarchy panel, delete the "Main Camera" as it is not required for AR (Fig. S.25 (a)).

b. Now get an AR Camera by selecting GameObject -> Vuforia Engine -> AR Camera (Fig. S.25 (b)).

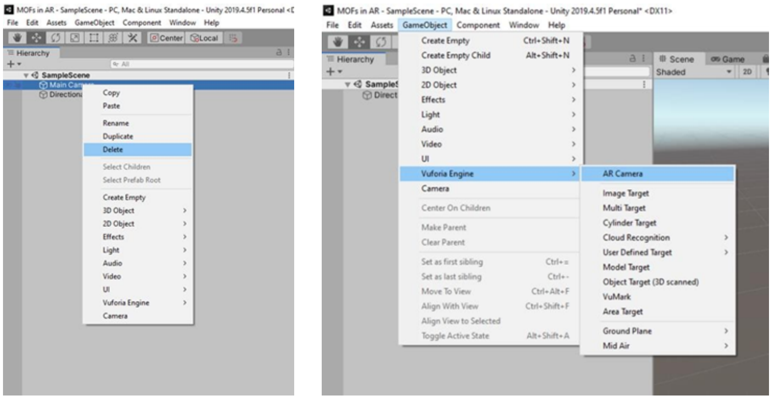

(a) (b)

Figure S.25. Setting up scene in Unity

c. Click on the AR Camera and on the Inspector window on the right, go to "Vuforia Behaviour (Script)" then to "Open Vuforia Engine Configuration" (Fig. S.26).

d. Paste the license key into the App License Key text box. This key was noted down earlier and can be found in the License Manager page on the Vuforia website.

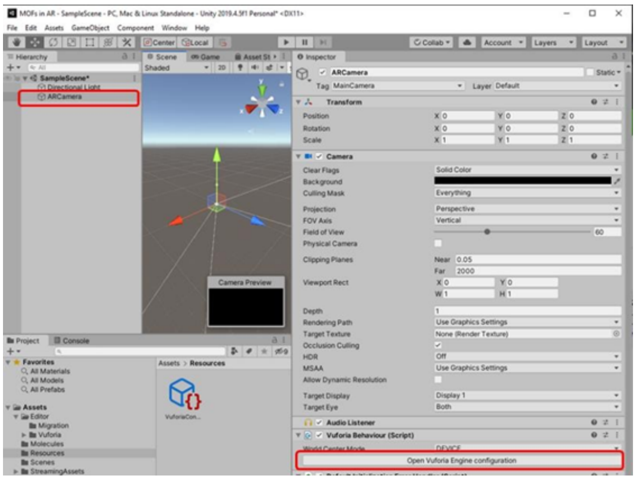

Figure S.26. Setting up AR camera in Unity

e. Go to GameObject -> Vuforia Engine -> Image Target (Fig.S.27). This should place an Image Target in the Scene (move this for now so that it is in a layer of its own and not a part of the AR Camera for example).

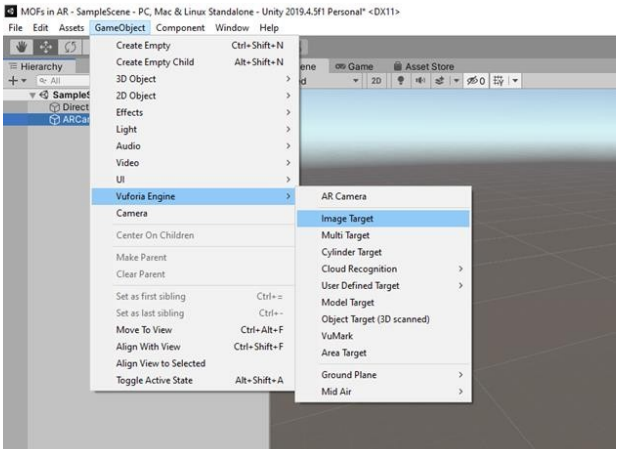

Figure S.27. Selecting image target step

f. Select this Image Target in the Hierarchy Panel and rename it to an appropriate MOF Target name.

g. Select this Image Target in the Hierarchy Panel and in the Inspector window navigate to "Image Target Behaviour (Script)" (Fig. S.28 (a)).

h. Click on Type and select "From Database" in the dropdown menu (Fig. S.28 (b)). Then pick the database and the desired target image from that database you want for this particular layer in the hierarchy. This will place the image target in the middle of the scene, use the middle scene editor window to place this target image to your liking, not overlapping other target images.

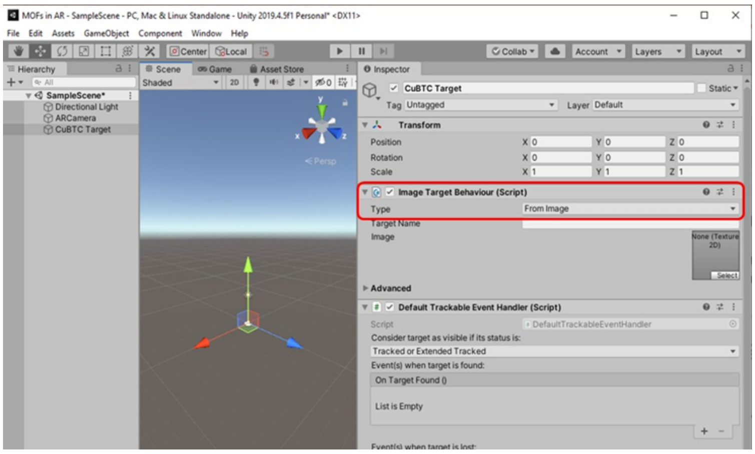

(a)

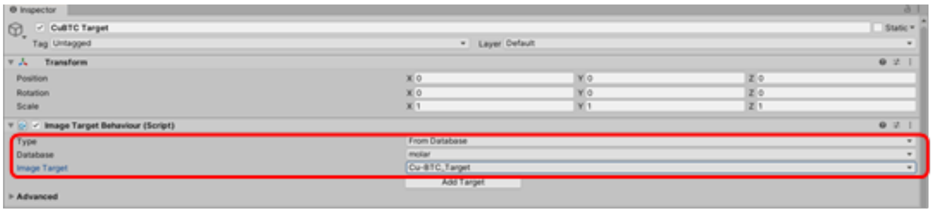

(b)

Figure S.28. Setting up process of target image

i. Now, select the MOF object from the molecules folder you wish to appear on top of the target image and drag it on to the scene. Position it using the scene editor and **scale it using the Inspector window** on the right hand side so that MOF object fits well over the target image. Note, double clicking on items in the Hierarchy panel can make locating target images, objects etc easier (Fig. S.29).

j. Use the individual axis to place the MOF. The middle mouse button moves the scene's camera position. The left mouse button moves and selects items and the right mouse button rotates the scene's camera.

k. Once happy with the setup in the Hierarchy panel, move the MOF object into the Target Image layer so that the object becomes a child of the target. Doing this earlier will make manipulating the model's position and scale relative to the target more difficult.

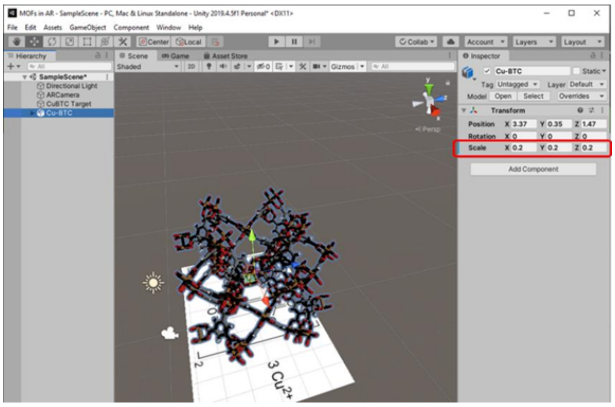

Figure S.29. The scene and scaling editor in Unity.

l. If using multiple MOFs with multiple targets, your hierarchy and objects should look similar to Fig. S.30.

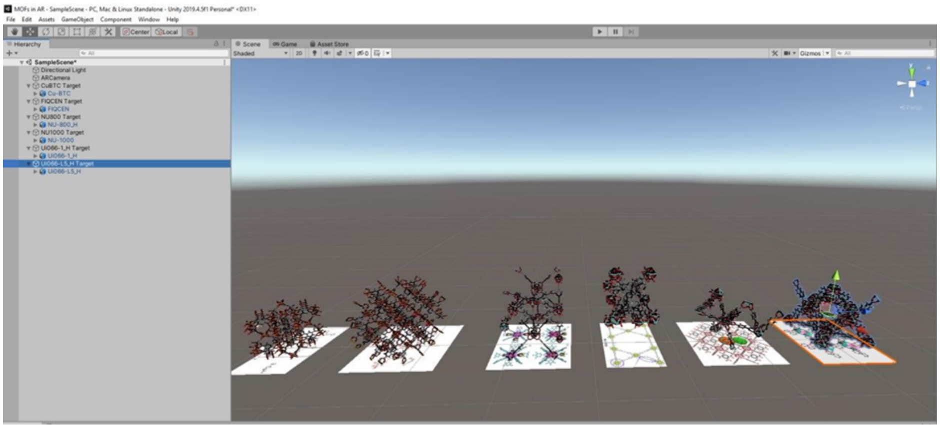

Figure S.30. Multiple MOFs with multiple targets

- Testing

a. If your device has a webcam, you could test the application before moving it to a smartphone. To do this, select AR Camera, go to “Open Vuforia Engine Configuration” in the Inspector window on the right and under “Play mode” select your webcam for the Camera Device (Fig. S.31(a)).

b. Press play in the main scene window and point your webcam at an example target image to see if it is recognised and an AR MOF is rendered over the target (Fig. S.31 (b)).

c. To finish the testing, press the play button again.

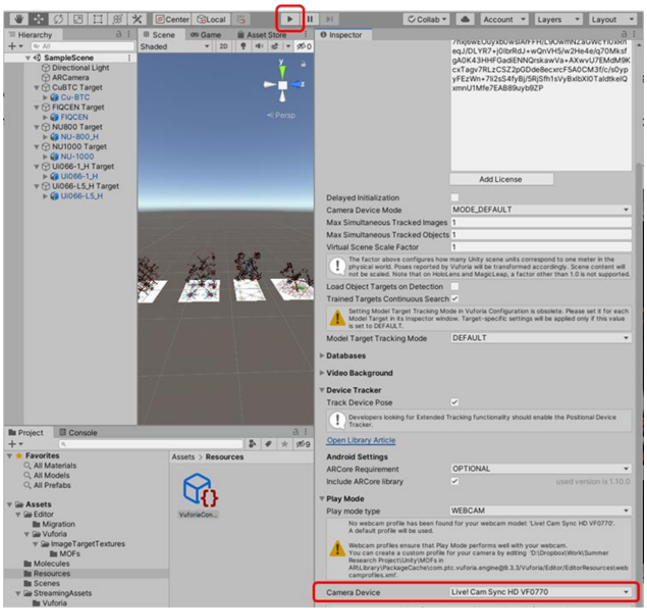

(a)

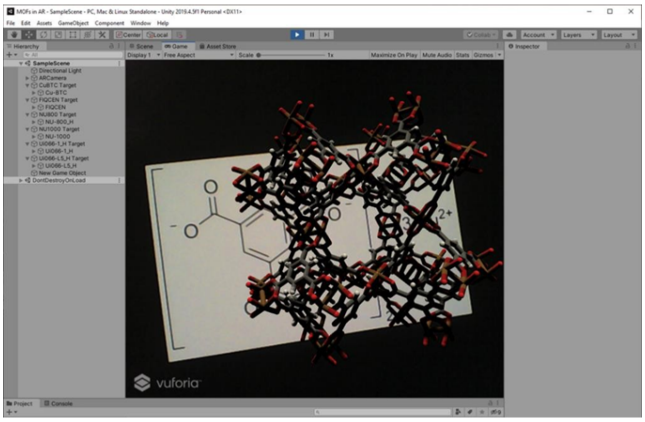

(b)

Figure S.31. Testing step of AR MOFs in Unity

- Building Android apps and testing on phones

a. It is possible to test this app on Android devices. To do this, enable developer options on your Android's settings by tapping on the "Build Number" several times until it grants you developer options. Then navigate back to developer options and enable USB debugging.

b. Connect your phone to your computer, wait for drivers to install if necessary, and open the project in Unity.

c. Navigate to File then "Build Settings" (Fig. S.32 (a)), then choose "Android" and select "Switch Platform" (Fig. S.32 (b)).

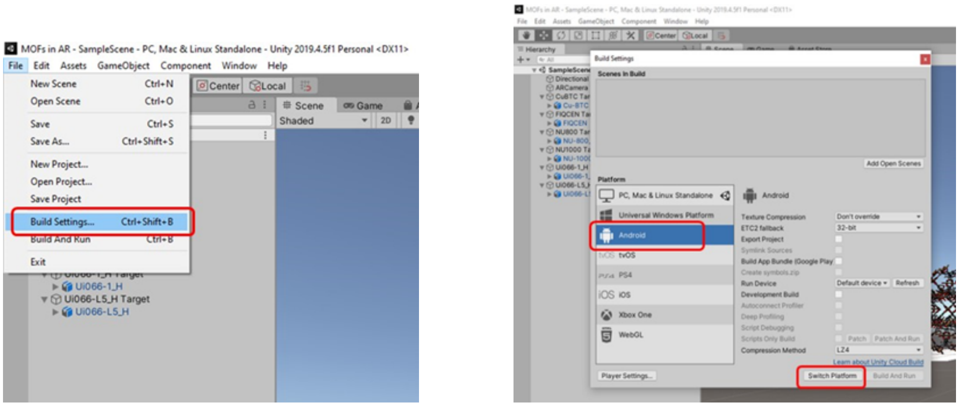

(a) (b)

Figure 24. Building android app step in Unity

d. Once Unity has finished this process, select Build and Run (Fig. S.33) which should compile an APK file onto your computer as well as push the application on to your phone and launch it once the process is finished. If a USB debugging prompt comes up on Unity, unlock your phone and click allow USB debugging from this computer and retry.

e. This APK can be shared and used to install the app on other Android devices. Apps made in Unity can also be published in the Play Store.

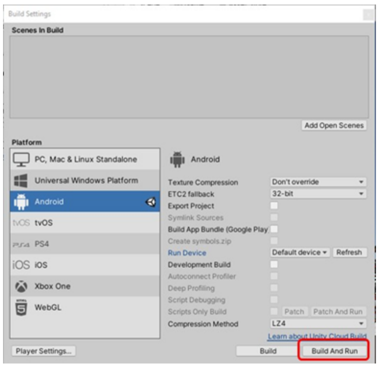

Figure 25. Compile an APK file step in Unity

For more detail and clear explanation, we provide video instruction that can be downloaded in this following link.

https://www.youtube.com/watch?time_continue=5&v=F_XjZ3Vr6IY

References.

(1) Moghadam, P. Z.; Li, A.; Wiggin, S. B.; Tao, A.; Maloney, A. G. P.; Wood, P. A.; Ward, S. C.; Fairen-Jimenez, D. Development of a Cambridge Structural Database Subset: A Collection of Metal–Organic Frameworks for Past, Present, and Future. *Chem. Mater.* **2017**, *29* (7), 2618–2625. https://doi.org/10.1021/acs.chemmater.7b00441.

(2) Katz, M. J.; Brown, Z. J.; Colón, Y. J.; Siu, P. W.; Scheidt, K. A.; Snurr, R. Q.; Hupp, J. T.; Farha, O. K. A Facile Synthesis of UiO-66, UiO-67 and Their Derivatives. *Chem. Commun.* **2013**, *49* (82), 9449. https://doi.org/10.1039/c3cc46105j.

(3) Dubbeldam, D.; Calero, S.; Ellis, D. E.; Snurr, R. Q. RASPA: Molecular Simulation Software for Adsorption and Diffusion in Flexible Nanoporous Materials. *Mol. Simul.* **2016**, *42* (2), 81–101. https://doi.org/10.1080/08927022.2015.1010082.

(4) Mayo, S. L.; Olafson, B. D.; Goddard, W. A. DREIDING: A Generic Force Field for Molecular Simulations. *J. Phys. Chem.* **1990**, *94* (26), 8897–8909. https://doi.org/10.1021/j100389a010.

(5) Rappe, A. K.; Casewit, C. J.; Colwell, K. S.; Goddard, W. A.; Skiff, W. M. UFF, a Full Periodic Table Force Field for Molecular Mechanics and Molecular Dynamics Simulations. *J. Am. Chem. Soc.* **1992**, *114* (25), 10024–10035. https://doi.org/10.1021/ja00051a040.

(6) Potoff, J. J.; Siepmann, J. I. Vapor–Liquid Equilibria of Mixtures Containing Alkanes, Carbon Dioxide, and Nitrogen. *AIChE J.* **2001**, *47* (7), 1676–1682. https://doi.org/10.1002/aic.690470719.

(7) Brunauer, S.; Emmett, P. H.; Teller, E. Adsorption of Gases in Multimolecular Layers. *J. Am. Chem. Soc.* **1938**, *60* (2), 309–319. https://doi.org/10.1021/ja01269a023.

(8) Gómez-Gualdrón, D. A.; Moghadam, P. Z.; Hupp, J. T.; Farha, O. K.; Snurr, R. Q. Application of Consistency Criteria To Calculate BET Areas of Micro- And Mesoporous Metal–Organic Frameworks. *J. Am. Chem. Soc.* **2016**, *138* (1), 215–224. https://doi.org/10.1021/jacs.5b10266.

(9) Rouquerol, J.; Llewellyn, P.; Rouquerol, F. Is the Bet Equation Applicable to Microporous Adsorbents? In *Characterization of Porous Solids VII*; Llewellyn, P. L., Rodriquez-Reinoso, F., Rouqerol, J., Seaton, N. B. T.-S. in S. S. and C., Eds.; Elsevier, 2007; Vol. 160, pp 49–56. https://doi.org/10.1016/S0167-2991(07)80008-5.

(10) Cavka, J. H.; Grande, C. A.; Mondino, G.; Blom, R. High Pressure Adsorption of CO_2 and CH_4 on Zr-MOFs. *Ind. Eng. Chem. Res.* **2014**, *53* (40), 15500–15507.

https://doi.org/10.1021/ie500421h.

(11) Cmarik, G. E.; Kim, M.; Cohen, S. M.; Walton, K. S. Tuning the Adsorption Properties of UiO-66 via Ligand Functionalization. *Langmuir* **2012**, *28* (44), 15606–15613. https://doi.org/10.1021/la3035352.

(12) Dovesi, R.; Erba, A.; Orlando, R.; Zicovich-Wilson, C. M.; Civalleri, B.; Maschio, L.; Rérat, M.; Casassa, S.; Baima, J.; Salustro, S.; Kirtman, B. Quantum-mechanical Condensed Matter Simulations with CRYSTAL. *WIREs Comput. Mol. Sci.* **2018**, *8* (4), e1360. https://doi.org/10.1002/wcms.1360.

(13) Manz, T. A.; Sholl, D. S. Chemically Meaningful Atomic Charges That Reproduce the Electrostatic Potential in Periodic and Nonperiodic Materials. *J. Chem. Theory Comput.* **2010**, *6* (8), 2455–2468. https://doi.org/10.1021/ct100125x.

(14) Moghadam, P. Z.; Li, A.; Liu, X.-W.; Bueno-Perez, R.; Wang, S.-D.; Wiggin, S. B.; Wood, P. A.; Fairen-Jimenez, D. Targeted Classification of Metal–Organic Frameworks in the Cambridge Structural Database (CSD). *Chem. Sci.* **2020**, *11* (32), 8373–8387. https://doi.org/10.1039/D0SC01297A.

(15) Eriksen, K.; Nielsen, B. E.; Pittelkow, M. Visualizing 3D Molecular Structures Using an Augmented Reality App. *J. Chem. Educ.* **2020**, *97* (5), 1487–1490. https://doi.org/10.1021/acs.jchemed.9b01033.

(16) Jmol. Jmol: an open-source Java viewer for chemical structures in 3D. http://jmol.sourceforge.net/. Accessed 25 Jul 2021.

(17) Unity. Unity 3d. Https://Unity.Com/. Accessed 25 Jul 2021.

(18) Vuforia. Vuforia. https://www.vuforia.com/. Accessed 25 Jul 2021.

(19) Moghadam, P. Z.; Li, A.; Wiggin, S. B.; Tao, A.; Maloney, A. G. P.; Wood, P. A.; Ward, S. C.; Fairen-Jimenez, D. Development of a Cambridge Structural Database Subset: A Collection of Metal–Organic Frameworks for Past, Present, and Future. *Chem. Mater.* **2017**, *29* (7), 2618–2625. https://doi.org/10.1021/acs.chemmater.7b00441.
